# Direct Oral Anticoagulant Levels at Time of Elective Surgery

**DOI:** 10.1001/jamanetworkopen.2025.55875

**Published:** 2026-02-04

**Authors:** Eleonora Camilleri, Payam Shahbabai, Mandana Rad, Charlotte A. Huijzer, Menno V. Huisman, Eveline L. A. van Dorp, Loes E. Visser, Henk-Jan Guchelaar, Suzanne C. Cannegieter, Nienke van Rein

**Affiliations:** 1 Department of Clinical Epidemiology, Leiden University Medical Center, Leiden, the Netherlands; 2 Department of Hospital Pharmacy, Haga Teaching Hospital, The Hague, the Netherlands; 3 Department of Anesthesiology, Haga Hospital Juliana Children’s Hospital, Den Haag, the Netherlands; 4 Department of Anesthesiology, Leiden University Medical Center, Leiden, the Netherlands; 5 Department of Internal Medicine, Division of Thrombosis and Hemostasis, Leiden University Medical Center, Leiden, the Netherlands; 6 Department of Hospital Pharmacy, Erasmus Medical Center, Rotterdam, the Netherlands; 7 Department of Clinical Pharmacy and Toxicology, Leiden University Medical Center, Leiden, the Netherlands; 8 Einthoven Laboratory for Vascular and Regenerative Medicine, Leiden University Medical Center, Leiden, the Netherlands

## Abstract

**Importance:**

Before elective surgery, direct oral anticoagulants (DOACs) are discontinued following a standardized protocol. However, this could result in insufficient lowering of DOAC levels that could increase bleeding risk.

**Objective:**

To estimate the proportion of patients with elevated DOAC levels at the time of elective surgery, evaluate factors associated with DOAC levels, and examine associated blood loss.

**Design, Setting, and Participants:**

This cohort study (DOAC Level Prior to Incision [DALI]) assessed adult patients prescribed a DOAC (apixaban, dabigatran, or rivaroxaban) for any indication and at any dose, undergoing an elective procedure requiring DOAC interruption between May 27, 2018, and February 25, 2024, at 2 Dutch hospitals.

**Exposure:**

Standardized interruption protocol (1 day before moderate- and 2 days before high bleeding-risk procedures) with interruption adjustments for the patient’s kidney function.

**Main Outcomes and Measures:**

Blood was drawn immediately before surgery to determine DOAC levels (by liquid chromatography–mass spectrometry). Proportions of preoperative DOAC levels of 30 ng/mL or higher and their 95% CIs were estimated, stratified by DOAC type and surgical bleeding risk. Factors associated with DOAC levels were identified through multivariable linear regression. Surgical blood loss and 30-day postoperative complications were described according to DOAC concentrations.

**Results:**

The study was terminated after including 257 patients (100 receiving apixaban, 100 receiving rivaroxaban, and 57 receiving dabigatran due to the slow inclusion rate of those receiving dabigatran; median [IQR] age, 72 [66-78] years; 173 male [67%]); 212 patients (82%) underwent a high bleeding-risk operation. Preprocedural DOAC levels were 30 ng/mL or higher in 7.6% (95% CI, 4.9%-11.6%) of patients. Dabigatran and rivaroxaban had similar proportions, whereas 13.1% (95% CI, 7.8%-21.2%) of patients treated with apixaban had levels of 30 ng/mL or higher. Treatment with apixaban, decreased kidney function, and a shorter interruption time were associated with higher levels. Surgical blood loss (median [range], 0 [0-4250] mL) was not associated with DOAC levels. Twelve patients (4.7%; 95% CI, 2.7%-8.0%), who all had DOAC levels less than 30 ng/mL, experienced major bleeding.

**Conclusions and Relevance:**

In this cohort study, most patients following the current protocol had DOAC levels less than 30 ng/mL, although the proportion of patients with elevated levels was higher for apixaban. Preoperative DOAC levels were not associated with blood loss during surgery.

## Key Points

**Question:**

What proportion of patients have elevated residual direct oral anticoagulant (DOAC) levels before an elective procedure when discontinuing treatment according to a standardized protocol (1 day before moderate bleeding-risk procedures and 2 days before high bleeding-risk procedures, with adjustments based on the patient’s kidney function)?

**Findings:**

In this cohort study of 257 patients, 7.6% had elevated DOAC levels of 30 ng/mL or higher before the procedure. Among those receiving apixaban, this proportion was higher (13.6%).

**Meaning:**

These results suggest that the standardized interruption protocol is associated with DOAC levels below 30 ng/mL, although the proportion of patients with levels of 30 ng/mL or greater was higher among those receiving apixaban.

## Teaser

This cohort study estimates the proportion of patients undergoing elective surgery who have elevated DOAC levels at the time of the procedure.

## Introduction

Perioperative management of direct oral anticoagulants (DOACs) in patients undergoing elective procedures is a daily challenge for clinicians.^1^ Approximately 10% to 30% of patients receiving oral anticoagulants yearly require temporary discontinuation for an elective procedure.^2,3^ Patients receiving DOAC are advised to discontinue treatment at a fixed period before the procedure without any laboratory monitoring.^4^ The discontinuation period is determined by the bleeding risk of the surgery, the patient’s kidney function, and the mean DOAC half-life.^4^ This advice is mainly based on the results of 2 cohort studies and a subanalysis of trials, which reported low rates of postoperative complications following a standardized protocol.^5,6,7^ Nonetheless, fixed discontinuation disregards the known interindividual variability in the half-life of DOACs. For instance, although the mean half-life of apixaban is 12 hours, its range is 10 to 20 hours, which could result in elevated preoperative DOAC levels in some individuals with a potentially associated increased bleeding risk.^8^ Previous studies included selected populations, evaluating mostly low- and moderate bleeding-risk procedures while not including patients with a higher burden of comorbidities, who may be at the highest risk of having elevated residual levels. Although up to 15% of patients had residual DOAC levels before surgery in these studies,^5,6^ only a few tested their association with clinical outcomes.^9,10^ Perioperative bleeding complications can lead to postoperative infections, longer hospital stays, worse clinical outcomes, and increased health care costs.^11^

For these reasons, our main aim was to estimate the proportion of patients with elevated DOAC levels at the time of a wide variety of elective procedures in an unselected population. We also aimed to estimate the proportion of patients with prolonged preoperative standard coagulation tests, investigate their specificity and sensitivity for preoperative DOAC levels, identify factors associated with preprocedural DOAC levels and coagulation tests, estimate the association of preprocedural DOAC levels with blood loss during surgery, and describe postprocedural complications.

## Methods

### Study Population

**Figure 1.**
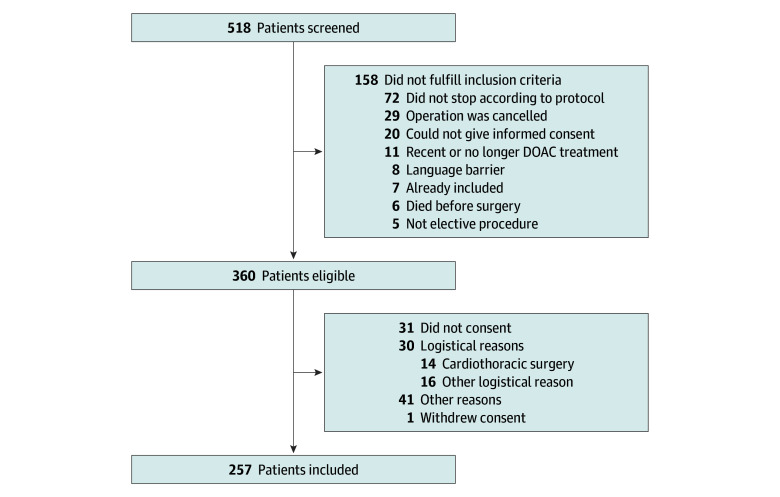
Flowchart of Inclusion in the DALI Study Logistical reasons included undergoing cardiothoracic surgery (the scheduling of which was not compatible with inclusion logistics) or unavailability of inclusion personnel on the day of the scheduled operation. Other reasons for noninclusion were related to challenges in screening and recruitment during the pandemic years. DALI indicates DOAC Level Prior to Incision; DOAC, direct oral anticoagulant.

The DOAC Level Prior to Incision (DALI) study was a cohort study of patients receiving DOACs who were undergoing an elective procedure between May 27, 2019, and February 25, 2024, in 2 Dutch hospitals (Leiden University Medical Center [LUMC] and Haga Teaching Hospital). All adult patients (aged ≥18 years) receiving a DOAC (apixaban, rivaroxaban, or dabigatran) who were scheduled for an elective procedure for which DOAC treatment needed to be interrupted were eligible for inclusion (planned sample size of 300 patients; see eMethods in Supplement 1 for details of sample size calculation). Patients receiving edoxaban were not included due to its infrequent use. Patients were not eligible if they were unable to give informed consent. Eligible patients were screened at preoperative outpatient clinics. They were contacted once the operation was planned and asked for written informed consent at least 24 hours before the procedure. For logistical reasons, patients undergoing cardiothoracic surgery could not be included. A total of 518 patients were screened for inclusion, of whom 360 (69%) were eligible. The main reason for noneligibility was that the perioperative protocol was not correctly followed (72 patients [14%]). Of the 360 eligible patients, 257 (71%) were included, and noninclusion was mainly due to logistical reasons (Figure 1). The study was terminated after reaching the planned sample size of 100 patients for apixaban and rivaroxaban, but due to the slow inclusion of patients receiving dabigatran, only 57 patients could be included. The DALI study was approved by the Medical Ethical Committee of the LUMC and adhered to the Strengthening the Reporting of Observational Studies in Epidemiology (STROBE) reporting guideline.

### Blood Collection and Measurements

Blood was collected on the day of the procedure by the anesthesiologist, shortly before the start of the operation, in a 2.7-mL 3.2% (109 mmol/L) sodium citrate tube, a 9-mL 3.2% sodium citrate tube, and a 3.5-mL tube, which contained silica particles. Following standard hospital procedures, prothrombin time (PT), activated partial thromboplastin time (aPTT), and creatinine levels were measured, and the estimated glomerular filtration rate (eGFR) was determined using the Chronic Kidney Disease Epidemiology formula. To obtain plasma for the measurement of DOAC concentrations, the 9-mL citrate tube was centrifuged in a centrifuge (Eppendorf 5810R, Eppendorf) for 8 minutes at 3000 relative centrifugal force at 22°C. The plasma was aliquoted and stored at −80 °C. DOAC levels were measured using high-performance liquid chromatography–mass spectrometry, with a lower limit of quantitation (LLOQ) of 15 ng/mL. Levels below this limit were quantifiable but not reliably measured.

### Data Collection

Baseline data (age, sex, DOAC type, dose, and indication) were collected from the electronic health records of the hospitals. Patients ceased DOAC intake according to the same protocol, based on national guidelines, in which DOAC doses were omitted 1 day before moderate bleeding-risk procedures and 2 days before high bleeding-risk procedures, with interruption intervals adjusted according to the patient’s kidney function (eTable 1 in Supplement 1).^12^ The exact time of intake of the last DOAC doses was recorded (eTable 2 in Supplement 1). Classification of the bleeding risk of surgery (moderate or high, as defined from the hospital protocol based on national guideline) (eTable 3 in Supplement 1) was derived from electronic records of the preoperative anesthesiology visit.^12^ Patients were followed up for 30 days after the procedure. Information on blood loss and blood products administration during surgery as well as the occurrence of major bleedings, infections, reoperation, and the use of blood products during follow-up were derived from the electronic health records. Bleeding complications were adjudicated according to the International Society on Thrombosis and Hemostasis classification for surgical major and minor bleeding complications, blinded for DOAC levels (by E.C. and N.v.R.).^13^

### Statistical Analysis

DOAC levels were described as median (IQR) and categorized as elevated if 30 ng/mL or higher. This cutoff was based on previous literature and on levels considered acceptable for a semiemergency operation by the hospital protocol.^5,6,12^ The proportions of patients with elevated levels and 95% CIs were estimated using the Wilson method and further stratified by type of DOAC and the bleeding risk of surgery.^14^

To estimate the proportion of patients with prolonged preoperative coagulation tests, PT and aPTT were described as median (IQR). PT was categorized as prolonged if 14.9 seconds or greater for patients included at the LUMC and 12.0 seconds or greater for patients included at the Haga Teaching Hospital. aPTT was categorized as prolonged if 31.7 seconds or greater for both centers (based on the reference ranges of the hospitals). These results were stratified by DOAC type, and the correlation with elevated DOAC levels was assessed using linear regression and the Spearman rank correlation coefficient (ρ). We calculated the sensitivity, specificity, positive predictive value (PPV), negative predictive value (NPV), and positive and negative likelihood ratios with 95% CIs of a normal PT and aPTT for identifying DOAC levels less than 30 ng/mL. These analyses were stratified by center (due to the use of different PT reagents).

Possible factors that could be used to identify patients with higher preoperative DOAC levels and coagulation tests were selected based on previous literature.^15,16^ Their association with DOAC levels, PT, and aPTT was assessed through univariable and multivariable regression (including all factors in the model), applying a Tobit model for DOAC levels to account for left-censoring at the LLOQ and linear regression for PT and aPTT. Continuous factors were inspected for nonlinear association with the outcomes.

We assessed the association between preoperative DOAC levels and surgical blood loss using linear (in milliliters) and logistic regression (any vs no blood loss), further adjusting and stratifying for bleeding risk of surgery. Major and minor bleeding events, infections, and reoperations occurring during follow-up were stratified by DOAC levels (<30 ng/mL or ≥30 ng/mL). The relative risks (RRs) and 95% CIs for these outcomes were estimated for patients with DOAC levels of 30 ng/mL or greater compared with levels less than 30 ng/mL.

As a sensitivity analysis, analyses using 2 different cutoffs for DOAC levels (15 ng/mL and 50 ng/mL) were performed. Analyses were performed using a complete-case approach because missing data on DOAC levels, PT, and aPTT occurred completely at random due to technical issues during blood collection or processing, using R studio, version 4.3.1 (R Foundation for Statistical Computing). A result was considered statistically significant when the 95% CI did not cross the null value.

## Results

### Study Population

The 257 included patients had a median (IQR) age of 72 (66-78) years, and 173 (67%) were male and 84 (33%) were female (Table 1). Rivaroxaban and apixaban were prescribed at the full dose in 72 (72%) and 87 (87%), respectively, whereas 36 patients receiving dabigatran (63%) received the full dose. Most procedures were classified as high bleeding risk (212 [82%]), and most were general and urological operations.

**Table 1.** Baseline Characteristics of the DALI Study Cohort at Baseline (Before the Elective Procedure)

Characteristic	No. (%) of patients^a^			
	Overall (N = 257)	Apixaban (n = 100)	Dabigatran (n = 57)	Rivaroxaban (n = 100)
Age, median (IQR), y	72 (66-78)	73 (65-78)	74 (69-78)	71 (64-76)
Sex				
Male	173 (67)	71 (71)	38 (67)	64 (64)
Female	84 (33)	29 (29)	19 (33)	36 (360)
Weight, median (IQR), kg	83 (75-97)	83 (75-96)	81 (76-98)	85 (75-98)
BMI, median (IQR)	27 (25-31)	27 (24-30)	27 (25-31)	28 (25-31)
Creatinine, median (IQR), mg/dL	0.90 (0.77-1.09)	0.95 (0.90-1.15)	0.89 (0.76-1.05)	0.87 (0.77-1.06)
eGFR, median (IQR), mL/min/1.73 m^2^	76 (59-86)	77 (58-86)	79 (62-86)	73 (57-85)
DOAC indication				
Atrial fibrillation	194 (75)	74 (74)	56 (98)	64 (64)
CHA_2_DS_2_-VASc score, median (IQR)	3 (2-4)	3 (2-4)	3 (2-4)	3 (2-4)
Venous thrombosis	41 (16)	16 (16)	0	25 (25)
Venous thrombosis due to cancer	11 (4)	6 (6)	0	5 (5)
ACS	5 (2)	1 (1)	1 (2)	3 (3)
CAD or PAD	3 (1)	1 (1)	0	2 (2)
Other	3 (1)	1 (1)	0	1 (1)
DOAC dose				
2.5 mg twice daily	NA	13 (13)	NA	4 (4)
5 mg twice daily	NA	87 (87)	NA	NA
110 mg twice daily	NA	NA	21 (37)	NA
150 mg twice daily	NA	NA	36 (63)	NA
10 mg once daily	NA	NA	NA	10 (10)
15 mg once daily	NA	NA	NA	14 (14)
20 mg once daily	NA	NA	NA	72 (72)
Surgical specialty				
Urology	60 (23)	24 (24)	10 (18)	26 (26)
General surgery	40 (16)	14 (14)	15 (26)	11 (11)
Gastrointestinal and liver surgery	24 (9)	11 (11)	5 (9)	8 (8)
Ear-nose-throat surgery	23 (9)	8 (8)	4 (7)	11 (11)
Orthopedics	21 (8)	9 (9)	4 (7)	8 (8)
Gynecology	17 (7)	5 (5)	4 (7)	8 (8)
Neurosurgery	14 (5)	6 (6)	2 (3)	6 (6)
Oncology	12 (5)	5 (5)	4 (7)	3 (3)
Vascular surgery	10 (4)	5 (5)	1 (2)	4 (4)
Plastic surgery	11 (4)	3 (3)	1 (2)	7 (7)
Radiology	7 (3)	3 (3)	4 (7)	0
Dermatology	3 (1)	1 (1)	0	2 (2)
Ophthalmology	3 (1)	0	1 (2)	2 (2)
Oral surgery	2 (1)	1 (1)	0 (0)	1 (1)
Other	10 (4)	5 (5)	2 (4)	3 (3)
Bleeding risk of surgery				
Moderate	45 (18)	21 (21)	13 (23)	11 (11)
High	212 (82)	79 (79)	44 (77)	89 (89)
DOAC interruption before elective procedure				
Time since last dose, h				
24	35 (14)	16 (16)	7 (12)	12 (12)
48	189 (74)	80 (80)	22 (39)	88 (88)
72	19 (7)	0	18 (32)	0
Other	14 (5)	4 (4)	10 (17)	0
Consultation with vascular nurse	154 (72)	53 (74)	34 (65)	65 (74)

Abbreviations: ACS, acute coronary syndrome; BMI, body mass index (calculated as weight in kilograms divided by height in meters squared); CAD, coronary artery disease; CHA_2_DS_2_-VASc, congestive heart failure, hypertension, age of 75 years or older (doubled), diabetes, stroke/transient ischemic attack (doubled), vascular disease, age of 65-74 years, and sex (female); DALI, DOAC Level Prior to Incision; DOAC, direct oral anticoagulant; eGFR, estimated glomerular filtration rate (calculated with the Chronic Kidney Disease Epidemiology formula); NA, not applicable; PAD, peripheral arterial disease.

SI conversion factor: To convert creatinine to micromoles per liter, multiply by 88.4.

^a^ Unless otherwise indicated.

### Preprocedural DOAC Levels

**Figure 2.**
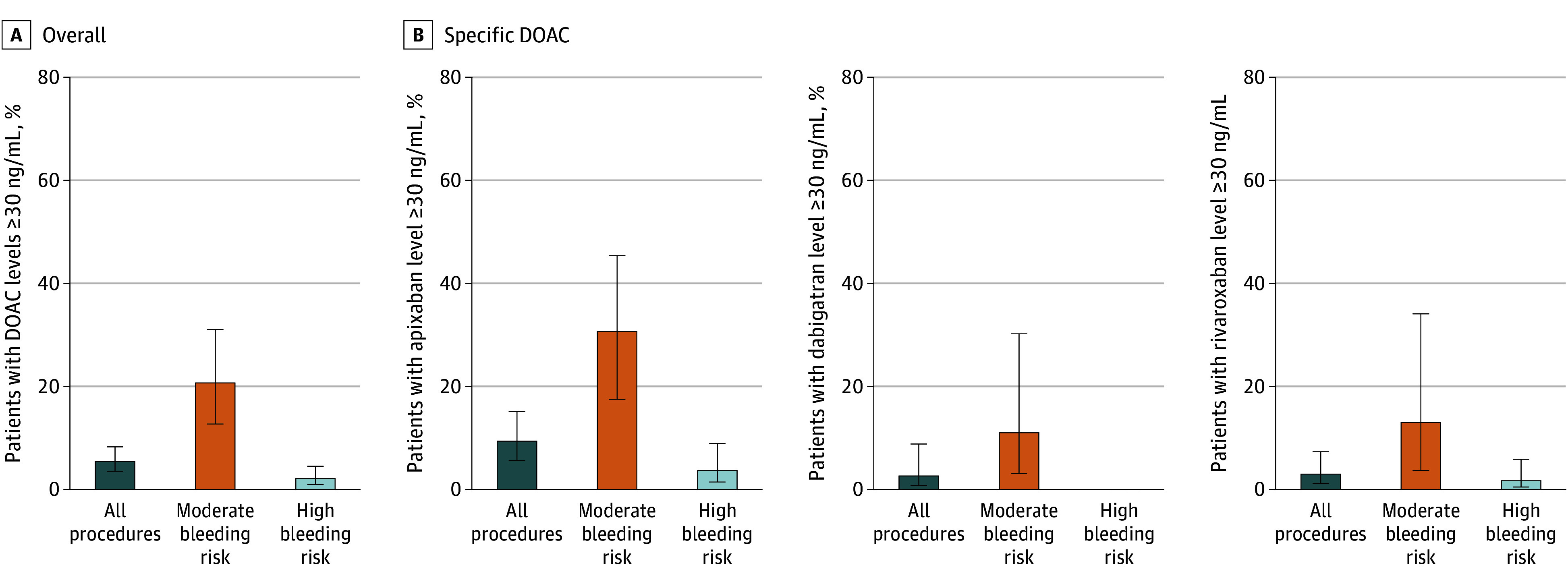
Proportion of Preoperative Direct Oral Anticoagulant (DOAC) Levels of 30 ng/mL or Greater for All Procedures and Stratified by Bleeding Risk of the Procedures For 7 patients, DOAC levels were missing due to technical issues during blood collection or processing. Error bars indicate 95% CIs.

Preprocedural DOAC levels were below the LLOQ (≤15 ng/mL) in 197 patients (77%). Median (IQR) preprocedural DOAC levels were 6.4 (1.3-12.3) ng/mL (eTable 4 in Supplement 1), and DOAC levels were 30 ng/mL or higher in 7.6% (95% CI, 4.9%-11.6%) of patients (Figure 2A). This proportion was similar for dabigatran (3.6%; 95% CI, 1.0%-12.3%) and rivaroxaban (4.2%; 95% CI, 1.6%-10.2%) (Figure 2B). In contrast, 13.1% (95% CI, 7.8%-21.2%) of patients treated with apixaban had levels of 30 ng/mL or greater. When stratified by the bleeding risk of the surgery, 2.9% (95% CI, 1.3%-6.2%) of patients undergoing a high bleeding-risk surgery had DOAC levels of 30 ng/mL or greater compared with 28.9% (95% CI, 17.7%-43.4%) of patients undergoing a moderate bleeding-risk operation (Figure 2A). Among the DOAC types, the proportion of patients with elevated levels undergoing a moderate bleeding-risk procedure was higher for patients receiving apixaban (42.9%; 95% CI, 24.5%-63.5%) (Figure 2B) than for patients receiving dabigatran (15.4%; 95% CI, 4.3%-42.2%) and rivaroxaban (18.2%; 95% CI, 5.1%-47.7%). Only 4 patients had an eGFR less than 30 mL/min/1.73 m^2^, of whom 2 had DOAC levels of 30 ng/mL or greater. Sensitivity analysis using different cutoffs of elevated DOAC levels yielded similar results (eFigure 1 and 2 in Supplement 1).

### Preprocedural PT and aPTT and Their Specificity and Sensitivity for Elevated DOAC Levels

The median (IQR) PT before surgery was 14.3 (12.3-15.3) seconds, and median (IQR) aPTT was 29.3 (27.0-31.6) seconds (eTable 4 in Supplement 1). Overall, 36.3% (95% CI, 30.3%-42.8%) and 23.1% (95% CI, 18.2%-29.0%) of patients had a prolonged PT and aPTT before surgery, respectively (eFigure 3A in Supplement 1). The proportion of patients with prolonged PT was similar across all 3 DOAC types (eFigure 3B in Supplement 1). Instead, aPTT was more frequently prolonged in patients receiving dabigatran (32.7%; 95% CI, 21.2%-46.6%) compared with those receiving apixaban (16.3%; 95% CI, 10.1%-25.2%) and rivaroxaban (25.0%; 95% CI, 17.1%-35.0%). Correlation of both PT and aPTT with preprocedural DOAC levels was low (ρ range, 0.1-0.5) (eFigures 4 and 5 in Supplement 1). Preoperative aPTT was weakly correlated with dabigatran levels (ρ = 0.5), whereas no correlation with PT and aPTT was observed for apixaban and rivaroxaban levels. For all DOACs, the sensitivity of a normal PT to identify patients with DOAC levels less than 30 ng/mL was moderate (ranging from 60.0%-67.5%) (eTable 5 in Supplement 1) with a good PPV (89.3%-96.6%). However, specificity (33.3%-50.0%) and NPV (3.6%-12.1%) were poor. Results of a normal aPTT were similar, except that a normal aPTT had a 100% PPV for dabigatran and rivaroxaban levels less than 30 ng/mL, with a statistically significant negative likelihood ratio. The analysis stratified by centers yielded similar results (eTable 6 and eFigures 6 and 7 in Supplement 1).

### Factors Associated With Preoperative DOAC Levels, PT, and aPTT

Undergoing high bleeding-risk surgery was associated with −33.6 ng/mL (95% CI, −44.5 to −22.7 ng/mL) lower preoperative DOAC levels compared with a moderate bleeding-risk surgery (Table 2). In line with this, patients with an interruption time of 48 hours or more had lower preoperative concentrations (−33.9 ng/mL [95% CI, −44.8 to −23.1 ng/mL]) compared with patients with a shorter interruption time. Treatment with dabigatran and rivaroxaban was associated with lower preoperative levels than apixaban (−26.7 ng/mL [95% CI, −43.0 to −10.4 ng/mL] and −18.2 ng/mL [95% CI, −30.1 to −6.2 ng/mL], respectively). In the model including all factors, treatment with apixaban, a lower eGFR, and a shorter interruption time remained associated with increased preoperative DOAC levels. No factor was associated with a preoperative PT or aPTT, except for the use of dabigatran, which was associated with a 1.4-second (95% CI, 0.1-2.7 seconds) prolongation of aPTT compared with apixaban.

**Table 2.** Factors Associated With Preoperative DOAC Levels, PT, and aPTT

Factor	Estimate (95% CI)					
	DOAC levels, ng/mL		PT, s		aPTT, s	
	Univariable	Multivariable^a^	Univariable	Multivariable^a^	Univariable	Multivariable^a^
Age, y	0.3 (−0.2 to 0.9)	0.4 (−0.1 to 0.9)	0.01 (−0.02 to 0.04)	0.01 (−0.02 to 0.04)	−0.01 (−0.05 to 0.04)	−0.03 (−0.1 to 0.03)
Sex, female vs male	3.5 (−7.9 to 14.9)	4.8 (−5.9 to 15.5)	−0.1 (−0.6 to 0.5)	−0.2 (−1.0 to 0.5)	−0.8 (−1.8 to 0.3)	−1.1 (−2.3 to 0.2)
Weight, kg	−0.1 (−0.4 to 0.2)	0.05 (−0.2 to 0.3)	−0.01 (−0.02 to 0.006)	−0.01 (−0.03 to 0.005)	0.003 (−0.02 to 0.03)	−0.02 (−0.05 to 0.02)
eGFR, mL/min/1.73 m^2^	−0.3 (−0.7 to 0.02)	−0.3 (−0.6 to −0.03)	−0.01 (−0.03 to 0.007)	−0.01 (−0.03 to 0.007)	- 0.01 (−0.04 to 0.02)	−0.02 (−0.06 to 0.01)
DOAC type						
Dabigatran vs apixaban	−26.7 (−43.0 to −10.4)	−28.4 (−44.2 to −12.7)	0.3 (−0.4 to 1.1)	0.5 (−0.3 to 1.4)	1.4 (0.1 to 2.7)	1.7 (0.2 to 3.1)
Rivaroxaban vs apixaban	−18.2 (−30.1 to −6.2)	−13.9 (−24.2 to −3.4)	0.1 (−0.5 to 0.7)	0.4 (−0.3 to 1.1)	0.4 (−0.7 to 1.5)	0.6 (−0.6 to 1.9)
DOAC dose, full dose vs reduced dose	11.1 (−2.5 to 24.7)	7.5 (−4.8 to 19.7)	0.4 (−0.3 to 1.0)	0.5 (−0.2 to 1.2)	0.5 (−0.6 to 1.7)	0.8 (−0.6 to 2.1)
DOAC interruption time, ≥48 vs <48 h	−33.9 (−44.8 to −23.1)	−23.7 (−48.8 to −1.5)	−0.2 (−0.9 to 0.6)	0.4 (−2.1 to 3.0)	−0.8 (−2.2 to 0.5)	0.5 (−4.2 to 5.1)
Bleeding risk of surgery, high vs moderate	−33.6 (−44.5 to −22.7)	−17.5 (−42.3 to 7.2)	−0.2 (−0.9 to 0.5)	−0.7 (−3.1 to 1.8)	−0.9 (−2.2 to 0.4)	−1.4 (−6.0 to 3.0)
Strong P-gp or CYP3A4 inhibitor	17.2 (−16.1 to 50.5)	7.2 (−20.5 to 35.1)	−0.1 (−2.4 to 2.3)	0.2 (−2.3 to 2.7)	−2.3 (−6.7 to 2.0)	−2.1 (−6.6 to 2.3)

Abbreviations: aPTT, activated partial thromboplastin time; CYP3A4, cytochrome P450 3A4; DOAC, direct oral anticoagulants; eGFR, estimated glomerular filtration rate; P-gp, P-glycoprotein; PT, prothrombin time.

^a^ Potential factors associated with preoperative DOAC levels, PT, and aPTT were selected based on prior literature.^15^^,^^16^ A Tobit model was applied for DOAC levels to account for left-censoring at the lower limit of quantitation (15 ng/mL) and linear regression for PT and aPTT. Continuous factors were inspected for nonlinear association with the outcomes. All factors were included in the multivariable model, irrespective of their association in the univariable model. Bleeding risk of surgery was defined according to eTable 3 in Supplement 1. Strong P-gp or CYP3A4 inhibitors were amiodarone, clarithromycin, cyclosporine, itraconazole, ketoconazole, quinidine, tacrolimus, tamoxifen, ritonavir, and verapamil. DOAC levels, PT, and aPTT were missing due to technical issues during blood collection or processing in 7, 10, and 6 patients, respectively.

### Surgical Blood Loss and Postoperative Complications

The median (IQR) surgical blood loss was 0 (0-100) mL (range, 0-4250 mL). Preoperative DOAC levels were not associated with an increase in surgical blood loss, although levels of 30 ng/mL or greater were associated with a slight increase in blood loss in moderate bleeding-risk procedures (Table 3). DOACs were restarted after a median (IQR) of 2 (2-4) days. A total of 35 patients (13.6%; 95% CI, 10.0%-18.3%) experienced bleeding during the 30 days of follow-up. In particular, 12 patients (4.7%; 95% CI, 2.7%-8%) had major bleeding, whereas 23 patients (8.9%; 95% CI, 6.0%-13.1%) had minor bleeding. Only 1 patient (5.3%; 95% CI, 1.0%-24.6%) with preoperative DOAC levels of 30 ng/mL or greater experienced any bleeding (RR, 0.4; 95% CI, 0.1-2.4) compared with patients with levels less than 30 ng/mL (Table 3). All 12 major bleeding events occurred in patients with DOAC levels less than 30 ng/mL (eTable 7 in Supplement 1). The postoperative period was complicated by an infection in 30 patients (11.6%; 95% CI, 8.3%-16.2%), which all occurred in patients with DOAC levels less than 30 ng/mL. An additional procedure was necessary in 21 patients (8.2%; 95% CI, 5.4%-12.2%), of whom 1 (5.3% 95% CI, 1.0%-24.6%) had a level of 30 ng/mL or greater. Sensitivity analysis using different cutoffs of elevated DOAC levels yielded similar results (eTables 8 and 9 in Supplement 1).

**Table 3.** Preprocedural DOAC Levels and Associated Surgical Blood Loss and Postoperative Complications

Complication	Estimates by DOAC levels					
	All procedures		Moderate bleeding risk		High bleeding risk	
	<30 ng/mL	≥30 ng/mL	<30 ng/mL	≥30 ng/mL	<30 ng/mL	≥30 ng/mL
Surgical blood loss						
Median (range), mL	0 (0 to 4250)	0 (0 to 1000)	0 (0 to 500)	0 (0 to 1000)	0 (0 to 4250)	0 (0 to 100)
β (95% CI)	1.0 [Reference]	−74.3 (−290.3 to 141.8)	1.0 [Reference]	119.1 (−1.1 to 239.3)	1.0 [Reference]	−198.7 (−604.6 to 207.3)
Adjusted β (95% CI)^a^	1.0 [Reference]	−3.7 (−236.2 to 228.8)	NA	NA	NA	NA
Any, No. (%)	80 (34.6)	6 (31.6)	9 (28.1)	5 (38.5)	71 (35.7)	1 (16.7)
RR (95% CI)	1.0 [Reference]	0.9 (0.5 to 1.8)	1.0 [Reference]	1.6 (0.4 to 6.2)	1.0 [Reference]	0.4 (0.1 to 2.2)
Adjusted RR (95% CI)^a^	1.0 [Reference]	0.9 (0.3 to 2.7)	NA	NA	NA	NA
All bleedings^b^						
No. (%)	34 (14.7)	1 (5.3)	4 (12.5)	1 (7.7)	30 (15.1)	0
RR (95% CI)	1.0 [Reference]	0.4 (0.1 to 2.4)	1.0 [Reference]	0.6 (0.1 to 5.0)	1.0 [Reference]	NA
Major bleeding^b^						
No. (%)	12 (5.2)	0	1 (3.1)	0	11 (5.5)	0
RR (95% CI)	1.0 [Reference]	NA	1.0 [Reference]	NA	1.0 [Reference]	NA
Infections^b^						
No. (%)	30 (11.0)	0	2 (6.2)	0 (0)	28 (14.1)	0
RR (95% CI)	1.0 [Reference]	NA	1.0 [Reference]	NA	1.0 [Reference]	NA
Reoperation^b^						
No. (%)	19 (8.2)	1 (5.3)	2 (6.2)	1 (7.7)	17 (8.5)	0
RR (95% CI)	1.0 [Reference]	0.6 (0.1 to 4.5)	1.0 [Reference]	1.2 (0.1 to 12.4)	1.0 [Reference]	NA

Abbreviations: DOAC, direct oral anticoagulants; NA, not applicable; RR, risk ratios.

^a^ From a model adjusted for the surgical bleeding risk (high or moderate).

^b^ Occurring in the 30 days of follow-up.

## Discussion

Despite being a frequent clinical challenge, perioperative management of DOACs remains underinvestigated.^17,18^ We investigated preprocedural DOAC levels in patients undergoing an elective procedure in 2 Dutch hospitals following the national standardized interruption protocol. Our main finding was that 7.6% of patients had preprocedural DOAC levels of 30 ng/mL or greater. For high bleeding-risk procedures, this proportion was below 3%, whereas approximately one-third of moderate bleeding-risk procedures (with shorter interruption time) had DOAC levels of 30 ng/mL or greater. Our findings are reassuring with respect to the overall efficacy of the standardized perioperative protocol in achieving low residual anticoagulant levels in clinicalpractice. This is in line with the PAUSE study, the largest study so far on perioperative DOAC management, in which DOAC levels of 30 ng/mL or greater were found in 20.6% of patients.^5^ The likely reason for our lower proportion of levels of 30 ng/mL or higher might be that we included mostly patients undergoing high bleeding-risk procedures (82% vs 33% in PAUSE). Accordingly, the incidence of major bleeding during follow-up was higher (4.7% [95% CI, 2.7%-8.0%] vs 1.4% [95% CI, 0.0%-2.0%] in PAUSE). Moreover, our population is mainly composed of male patients with an eGFR of 50 mL/min/1.73 m^2^ or greater, whereas female sex and eGFR less than 50 mL/min/1.73 m^2^ were previously identified as predictors of elevated residual preprocedural levels, although the association with female sex was not confirmed by our findings.^15^ Of interest, the type of DOAC, lower eGFR, and the interruption time had the strongest associations with preoperative DOAC levels. A higher proportion of patients treated with apixaban had levels 30 ng/mL or greater in our study, compared with patients receiving rivaroxaban or dabigatran, which remained in the multivariable model. In the PAUSE study, apixaban use was also associated with a higher likelihood of levels 30 ng/mL or greater before moderate bleeding-risk procedures.^15^ This consistent result may indicate that prolonging the interruption time or adjusting it using parameters beyond kidney function and surgical bleeding risk is necessary to achieve levels less than 30 ng/mL.

In addition to DOAC levels, we evaluated the preprocedural PT and aPTT. Our results support the insufficient utility of routine coagulation tests to assess elevated residual DOAC levels. Preoperative PT and aPTT were frequently abnormal and not correlated with preoperative DOAC levels, with low specificity and NPV, similar to previous results.^19^ The high PPV (>90%) for a normal PT and aPTT lend support for their use to exclude residually elevated levels of dabigatran and rivaroxaban, as recommended by national guidelines.^12^ Only aPTT was weakly correlated with preoperative dabigatran levels and showed better sensitivity in line with the literature.^20^ We did not observe a correlation of PT with rivaroxaban levels, while PT is normally more sensitive for rivaroxaban than apixaban.^21^

The perioperative setting remains a potential indication for DOAC plasma level monitoring.^22,23^ We defined elevated residual DOAC levels as 30 ng/mL or higher, but a safe preprocedural DOAC concentration threshold is not known. Future studies should focus on defining such a threshold, which will inform the definition of a safe preprocedural concentration. Current knowledge is mostly based on expert consensus and few studies that evaluated associations between preprocedural DOAC levels and periprocedural outcomes.^9,10^ We did not find an association between DOAC levels and surgical blood loss in unadjusted and adjusted analysis, although a slight increase in blood loss was observed in moderate bleeding-risk procedures. Given that one-third of patients undergoing these procedures had levels 30 ng/mL or greater, this may indicate the need to consider a longer interruption period. However, it remains unclear whether this modest increase in blood loss is clinically relevant, as this likely depends on the specific procedure. Decisions regarding interruption time must be carefully balanced against the potential increase in thrombotic risk, which is currently unknown. Moreover, blood loss greatly differs between operations within the same bleeding-risk category (eg, surgery of conjunctiva compared to major oncological operations, both classified as high bleeding risk). A refinement of these categories, which might not fully capture the extent of surgical blood loss, is necessary to truly assess this association. Our study lacked statistical power to assess the association of DOAC levels with bleeding events during follow-up. Of note, all major bleeding events occurred in patients with DOAC levels less than 30 ng/mL. Of the perioperative context, studies reported associations of DOAC levels with both bleeding and thromboembolic events.^24,25,26,27,28,29^ Recent studies showed an influence of residual DOAC levels on thrombin generation, which mediated the risk of postoperative bleeding complications.^9,30,31^ Altogether, larger studies are necessary to identify a relationship between DOAC levels and perioperative blood loss, considering operations with similar blood loss. Elevated DOAC levels might still be associated with unfavorable outcomes in specific high bleeding-risk operations.

### Strengths and Limitations

Strengths of our study include the unselected population of DOAC users undergoing a wide range of elective procedures. We mainly included patients undergoing procedures with high bleeding risk, which provides reassurance on the safety of the current protocol in achieving overall low DOAC preoperative concentrations during a high bleeding-risk setting. DOAC levels were measured with high-performance liquid chromatography–mass spectrometry, the current gold standard with a relatively low LLOQ. Missing data were less than 3% and no patient was lost to follow-up.

This study has limitations. We were unable to complete enrollment for dabigatran-treated patients and terminated the study after recruiting 257 patients of a planned 300. Study recruitment was severely impacted by the COVID-19 pandemic, as inclusions were temporarily halted during the lockdowns and the number of elective operations markedly decreased. In addition, the use of dabigatran decreased in the region over time. Second, we were able to include 71% of eligible patients; however, noninclusion was mainly at random (eg, due to logistical reasons) thus unlikely to cause bias. Third, because our study population mainly consisted of patients undergoing high bleeding-risk procedures at an academic center and a large teaching hospital, our results may not be generalizable to centers that primarily perform moderate bleeding-risk operations. Lastly, we lacked sufficient statistical power to estimate the association between preoperative DOAC levels and postoperative complications, as sample size was based on estimating the proportion of patients with elevated DOAC levels.

## Conclusion

In this cohort study, preoperative DOAC levels were 30 ng/mL or greater in 7.6% of patients, and this proportion was 2.9% for patients undergoing high bleeding-risk procedures and 28.9% for patients undergoing moderate bleeding-risk procedures. Treatment with apixaban, a lower eGFR, and a shorter interruption time were associated with an increase in preoperative DOAC concentrations. Major bleeding events during follow-up occurred only in patients with levels less than 30 ng/mL, and an association between DOAC levels and surgical blood loss was not observed. Although our results support the overall safety of the current protocol, extending the interruption of apixaban by an additional 12 hours could be considered, given the higher proportion of patients receiving apixaban with levels of 30 ng/mL or greater.

## Notes

Supplement 1. **eMethods.** Sample size considerations **eTable 1.** Perioperative protocol for standardized period of interruption of DOAC before an elective procedure **eTable 2.** Median time (in hours) between the last DOAC dose and start of the elective procedure, stratified by DOAC **eTable 3.** Classification of surgical bleeding risk **eTable 4.** Median and interquartile of DOAC levels, PT and aPTT, further stratified by DOAC type **eTable 5.** Sensitivity and specificity of PT and aPTT for DOAC levels <30 ng/mL **eTable 6.** Sensitivity and specificity of PT and aPTT for DOAC levels <30 ng/mL, stratified by center of inclusion **eTable 7.** DOAC levels of patients experiencing a major bleeding during follow-up **eTable 8.** Sensitivity analysis on preprocedural DOAC levels (using an alternative cut-off of 15 ng/mL) and associated surgical blood loss and postoperative complications **eTable 9.** Sensitivity analysis on preprocedural DOAC levels (using an alternative cut-off of 50 ng/mL) and associated surgical blood loss and postoperative complications **eFigure 1.** Sensitivity analysis on the proportion of preoperative DOAC levels ≥15 ng/mL overall (A) and by specific DOAC (B), shown for all procedures and stratified by bleeding risk of the procedures **eFigure 2.** Sensitivity analysis on the proportion of preoperative DOAC levels ≥50 ng/mL overall (A) and by specific DOAC (B), shown for all procedures and stratified by bleeding risk of the procedures **eFigure 3.** Proportion of preoperative prolonged PT and aPTT levels for all DOACs (A) and stratified by DOAC type (A) **eFigure 4.** Correlation between preoperative PT and DOAC levels (A. all DOACs, B. stratified by DOAC type) **eFigure 5.** Correlation between preoperative aPTT and DOAC levels (A. all DOACs, B. stratified by DOAC type **eFigure 6.** Correlation between preoperative PT and DOAC levels, stratified by center (A. all DOACs, B. stratified by DOAC type) **eFigure 7.** Correlation between preoperative aPTT and DOAC levels, stratified by center (A. all DOACs, B. stratified by DOAC type)

Supplement 2.
Data Sharing Statement
