# Supplementary material for: Direct Oral Anticoagulant Levels at Time of Elective Surgery
Source: JAMA Netw Open. 2026 Feb 4;9(2):e2555875. doi: 10.1001/jamanetworkopen.2025.55875 (PMC12873767; doi:10.1001/jamanetworkopen.2025.55875)
Supplement: Supplement 1. — eMethods. Sample size considerations eTable 1. Perioperative protocol for standardized period of interruption of DOAC before an elective procedure eTable 2. Median time (in hours) between the last DOAC dose and start of the elective procedure, stratified by DOAC eTable 3. Classification of surgical bleeding risk eTable 4. Median and interquartile of DOAC levels, PT and aPTT, further stratified by DOAC type eTable 5. Sensitivity and specificity of PT and aPTT for DOAC levels <30 ng/mL eTable 6. Sensitivity and specificity of PT and aPTT for DOAC levels <30 ng/mL, stratified by center of inclusion eTable 7. DOAC levels of patients experiencing a major bleeding during follow-up eTable 8. Sensitivity analysis on preprocedural DOAC levels (using an alternative cut-off of 15 ng/mL) and associated surgical blood loss and postoperative complications eTable 9. Sensitivity analysis on preprocedural DOAC levels (using an alternative cut-off of 50 ng/mL) and associated surgical blood loss and postoperative complications eFigure 1. Sensitivity analysis on the proportion of preoperative DOAC levels ≥15 ng/mL overall (A) and by specific DOAC (B), shown for all procedures and stratified by bleeding risk of the procedures eFigure 2. Sensitivity analysis on the proportion of preoperative DOAC levels ≥50 ng/mL overall (A) and by specific DOAC (B), shown for all procedures and stratified by bleeding risk of the procedures eFigure 3. Proportion of preoperative prolonged PT and aPTT levels for all DOACs (A) and stratified by DOAC type (A) eFigure 4. Correlation between preoperative PT and DOAC levels (A. all DOACs, B. stratified by DOAC type) eFigure 5. Correlation between preoperative aPTT and DOAC levels (A. all DOACs, B. stratified by DOAC type eFigure 6. Correlation between preoperative PT and DOAC levels, stratified by center (A. all DOACs, B. stratified by DOAC type) eFigure 7. Correlation between preoperative aPTT and DOAC levels, stratified by center (A. all DOACs, B. stratified [file jamanetwopen-e2555875-s001.pdf]

Supplemental Online Content

Camilleri E, Shahbabai P, Rad M, et al. Direct oral anticoagulant levels at time of elective surgery: the DALI study. *JAMA Netw Open*. 2026;9(2):e2555875. doi:10.1001/jamanetworkopen.2025.55875

Methods. Sample size considerations

eTable 1. Perioperative protocol for standardized period of interruption of DOAC before an elective procedure

eTable 2. Median time (in hours) between the last DOAC dose and start of the elective procedure, stratified by DOAC

eTable 3. Classification of surgical bleeding risk

eTable 4. Median and interquartile of DOAC levels, PT and aPTT, further stratified by DOAC type

eTable 5. Sensitivity and specificity of PT and aPTT for DOAC levels <30 ng/mL

eTable 6. Sensitivity and specificity of PT and aPTT for DOAC levels <30 ng/mL, stratified by center of inclusion

eTable 7. DOAC levels of patients experiencing a major bleeding during follow-up

eTable 8. Sensitivity analysis on preprocedural DOAC levels (using an alternative cut-off of 15 ng/mL) and associated surgical blood loss and postoperative complications

eTable 9. Sensitivity analysis on preprocedural DOAC levels (using an alternative cut-off of 50 ng/mL) and associated surgical blood loss and postoperative complications

eFigure 1. Sensitivity analysis on the proportion of preoperative DOAC levels ≥15 ng/mL overall (A) and by specific DOAC (B), shown for all procedures and stratified by bleeding risk of the procedures

eFigure 2. Sensitivity analysis on the proportion of preoperative DOAC levels ≥50 ng/mL overall (A) and by specific DOAC (B), shown for all procedures and stratified by bleeding risk of the procedures

eFigure 3. Proportion of preoperative prolonged PT and aPTT levels for all DOACs (A) and stratified by DOAC type (A)

eFigure 4. Correlation between preoperative PT and DOAC levels (A. all DOACs, B. stratified by DOAC type)

eFigure 5. Correlation between preoperative aPTT and DOAC levels (A. all DOACs, B. stratified by DOAC type

eFigure 6. Correlation between preoperative PT and DOAC levels, stratified by center (A. all DOACs, B. stratified by DOAC type)

eFigure 7. Correlation between preoperative aPTT and DOAC levels, stratified by center (A. all DOACs, B. stratified by DOAC type)

This supplemental material has been provided by the authors to give readers additional information about their work.

© 2026 Camilleri E et al. *JAMA Network Open.*

eMethods. Sample size considerations

We planned to include a total of 100 patients for each DOAC (300 patients in total). Based on previous research [1,2], this would yield 45 patients with elevated DOAC levels and conserving an α of 0.05 to observe up to 15% of patients with elevated DOAC levels with a 95% CI of 11% to 19%, which was considered an acceptable CI.

eReferences

1. Douketis JD, Spyropoulos AC, Duncan J, et al. Perioperative Management of Patients With Atrial Fibrillation Receiving a Direct Oral Anticoagulant. *JAMA Intern Med*. Aug 5 2019;doi:10.1001/jamainternmed.2019.2431

2. Douketis JD, Wang G, Chan N, et al. Effect of standardized perioperative dabigatran interruption on the residual anticoagulation effect at the time of surgery or procedure. *J Thromb Haemost*. Jan 2016;14(1):89-97. doi:10.1111/jth.13178

© 2026 Camilleri E et al. *JAMA Network Open.*

eTable 1. Perioperative protocol for standardized period of interruption of DOAC before an elective procedure

The protocol is based on the bleeding risk of the surgery, the renal function (measured by eGFR) and the average DOAC half-life. Apixaban and dabigatran are taken twice daily; stopping 24 hours before the procedure therefore means the last dose is taken on the morning of day -1 (procedure on day 0). Rivaroxaban is taken once daily; if taken in the morning, stopping 24 hours before the procedure means the last dose is taken on the morning of day -1 (procedure on day 0). If taken in the evening, stopping 24 hours before the procedure means the last dose is taken on the evening of day -2. (DOAC, directed oral anticoagulants; eGFR, estimated glomerular filtration rate.)

		Time of last dose before procedure	
**DOAC**	**eGFR (mL/min)**	**Moderate bleeding risk**	**High bleeding risk**
**Apixaban**	> 30	24 hours	48 hours
	< 30	36 hours	48 hours
**Rivaroxaban**	> 30	24 hours	48 hours
	< 30	36 hours	48 hours
**Dabigatran**	> 80	24 hours	48 hours
	50 – 80	36 hours	72 hours
	30 – 50	48 hours	96 hours

© 2026 Camilleri E et al. *JAMA Network Open.*

eTable 2. Median time (in hours) between the last DOAC dose and start of the elective procedure, stratified by DOAC

Elective procedures	Apixaban	Dabigatran		Rivaroxaban	
	Median (IQR)	Median (IQR)	Mean difference vs Apixaban*	Median (IQR)	Mean difference vs. Apixaban*
**All**	50.1 (48.0 to 52.9)	60.9 (48.5 to 74.1)	11.1 (5.9 to 16)	51.0 (47.9 to 61.8)	3.5 (-0.9 to 7.9)
**Moderate bleeding risk**	29.5 (25.0 to 39.0)	35.8 (28.5 to 40.2)	3.7 (-4.4 to 11.8)	27.0 (26.3 to 38.4)	-1.7 (-10.2 to 6.8)
**High bleeding risk**	51.2 (49.0 to 53.2)	72.0 (51.2 to 75.6)	13.6 (8.6 to 18.6)	52.2 (48.5 to 62.1)	1.8 (-2.2 to 5.9)

*Mean differences in hours between the last dose and the start of the elective procedures compared with apixaban.

© 2026 Camilleri E et al. *JAMA Network Open.*

eTable 3. Classification of surgical bleeding risk

	Moderate bleeding risk	High bleeding risk
**Anesthesiology**		- Epidural
**Biopsies and punctures**	- Bone marrow biopsy<br>- Ascites drainage<br>- Pleural drainage	- Lumbar puncture
**Cardiology**	- Pericardial puncture and drainage<br>- Mitral valve dilatation<br>- Pacemaker/ Implantable Cardioverter-Defibrillators implantation	
**Dermatology**	- Mole surgery<br>- Local skin plastic and skin grafts	
**Gynecology**	- All procedures that cannot be classified as low or high bleeding risk	- Debulking<br>- Myoma nucleation<br>- Radical uterine extirpation<br>- Exenteration<br>- Staging Ovary/Endometrial Carcinoma

© 2026 Camilleri E et al. *JAMA Network Open.*

**General Surgery**	- Open cholecystectomy<br>- Hemorrhoids surgery<br>- Axillary lymph node dissection<br>- Laparoscopic surgery	- Vascular surgery<br>- Head/neck surgery, abdominal surgery and general surgery > 45 minutes<br>- Major Oncological Surgery<br>- Kidney transplant<br>- Liver/Pancreas/Spleen Surgery
**Ear, Nose and Throat Surgery**	- Tonsillectomy<br>- Conchotomy<br>- Tongue/floor of mouth resection<br>- Pan endoscopy + biopsy<br>- Bone Conduction Implant<br>- Meatoplasty<br>- Tympanic tube surgery	- Septal correction<br>- Sinus surgery<br>- Rhinoplasty<br>- Head/neck soft tissue surgery (lateral/median cervical cyst, command resection, laryngectomy, parotid, submandibular gland, cervical lymph node dissection)<br>- Myringoplasty / Middle Ear Inspection<br>- Chronic Inflammation/Cholesteatoma Remediation, Cochlear Implant<br>- Stapedotomy<br>- Translabyrinthine Bridge Angle Surgery<br>- Vocal cords surgery
**Lung diseases**	- Pleural drainage	- Bronchoscopy with or without biopsies

© 2026 Camilleri E et al. *JAMA Network Open.*

**Gastroenterology and Hepatology**		- Colonoscopy with possibly polypectomy<br>- Polypectomy, mucosal resection, sclerosing<br>- Endoscopic Retrograde Cholangiopancreatography with papillotomy, sphincterotomy, crush/basket extraction<br>- Endo-ultrasound with fine needle aspiration<br>- Percutaneous endoscopic gastrostomy placement<br>- Laser ablation, Argon plasma coagulation<br>- Treatment (sclerosis) esophageal varices<br>- Esophageal expansion by savary dilators/balloon technique<br>- Esophageal stent placement<br>- Therapeutic double balloon enteroscopy<br>- Liver biopsy
**Oral Diseases, Maxillofacial and Maxillofacial Surgery**	- Wisdom Tooth Extraction	
**Neurosurgery**	- Carpal tunnel correction	- Intracranial and Spinal Surgery
**Ophthalmology**		- Endoscopic dacryocystorhinostomy<br>- Orbital operations<br>- Trabeculectomy<br>- Conjunctiva surgeries only on indication (excision, malignancy or major procedure)

© 2026 Camilleri E et al. *JAMA Network Open.*

**Orthopedics / Traumatology**	- Shoulder, Foot and Hand Surgery<br>- Arthroscopy	- Knee and hip replacement<br>- Open Vertebral Surgery
**Plastic surgery**	- Skin cancer excision	- Reconstructive Plastic Surgery
**Radiology**	- Intra-arterial procedures<br>- Drainage fluid collections and removal of drainage impediment (e.g., nephrostomy, abscess drainage, ascites drainage, including switches)<br>- JJ catheter<br>- Tunneled venous lines<br>- Percutaneous endoscopic gastrostomy switch<br>- Bubbling	- Ultrasound/Computer tomography-guided deep organ biopsy (puncture site impressions not possible), kidney biopsy, liver biopsy, bone biopsy, some soft-week tumor biopsy<br>- Radio Frequency Ablation Deep Organ<br>- Percutaneous endoscopic gastrostomy placement<br>- Cementation (hip replacement and vertebra)<br>- Transjugular Intrahepatic Portosystemic Shunt.<br>- Islet transplantation via vena portae<br>- Porta-embolization<br>- Aneurysmal bone cyst Embolization<br>- Ever's Thoracal<br>- Lumbar puncture

© 2026 Camilleri E et al. *JAMA Network Open.*

**Urology**	- JJ-placement (retrograde)	- Ureteroscopy/Ureterorenoscopy (endourology)<br>- Prosthesiology (penis/sphincter)<br>- Sling Surgery<br>- Laparoscopic kidney/ureter surgery<br>- Robot Assisted Bladder/Kidney/Ureters Surgery<br>- Transurethral procedures (bladder/prostate)<br>- Intravesical Botox<br>- Scrotal surgery (inguinal and scrotal approach)<br>- Peniel surgery<br>- Open Abdominal Surgery<br>- Prostate biopsies transrectal<br>- Suprapubic catheter placement

© 2026 Camilleri E et al. *JAMA Network Open.*

eTable 4. Median and interquartile of DOAC levels, PT and aPTT, further stratified by DOAC type

PT and aPTT are further stratified by DOAC levels (<30 ng/mL, or ≥30 ng/mL). PT, prothrombin time; aPTT, activated thromboplastin time; DOAC, directed oral anticoagulants.

Median (IQR)			**Overall**	**Apixaban**	**Dabigatran**	**Rivaroxaban**
**DOAC levels**, ng/mL			6.4<br>(1.3 to 12.3)	7.5<br>(5.2 to19.7)	5.6<br>(1.6 to 8.1)	2.3<br>(0 to 9.3)
**PT**, s	overall		14.3<br>(12.4 to 15.3)	14.2<br>(11.5 to 15.2)	14.5<br>(13.0 to 15.4)	14.3<br>(12.9 to 15.3)
	Stratified by DOAC					
		<30 ng/mL	14.3<br>(12.5 to 15.3)	14.2<br>(11.9 to 15.3)	14.4<br>(12.9 to 15.3)	14.3<br>(13.1 to 15-2)
		≥30 ng/mL	14.5<br>(11.2 to 15.5)	14.2<br>(11.6 to 15.2)	15.9<br>(15.2 to 16.7)	11.2<br>(11.0 to 14.5)
**aPTT**, s	overall		29.3<br>(27.0 to 31.6)	29.0<br>(27.0 to 31.2)	30.6<br>(27.0 to 33.0)	29.5<br>(26.7 to 31.7)
	Stratified by DOAC					
		<30 ng/mL	29.3<br>(27.0 to 31.4)	28.9<br>(27.0 to 31.0)	30.3<br>(27.0 to 32.6)	29.4<br>(26.6 to 31.3)
		≥30 ng/mL	31.2<br>(29.4 to 34.3)	29.9<br>(29.9 to 31.2)	39.7<br>(35.8 to 43.5)	35.0<br>(33.5 to 37.0)

DOAC levels were missing for 7 patients, aPTT for 6 patients, PT for 10 patients (due to issue with blood collection or processing)

© 2026 Camilleri E et al. *JAMA Network Open.*

eTable 5. Sensitivity and specificity of PT and aPTT for DOAC levels <30 ng/mL

Sensitivity: Proportion of patients with DOAC levels <30 ng/mL with normal PT or aPTT; Specificity: Proportion of patients with DOAC levels ≥30 ng/mL with a prolonged PT or aPTT; Positive predictive value (PPV): Proportion of patients with a normal PT or aPTT who have a DOAC level <30 ng/mL; Negative predictive value (NPV): Proportion of patients with a prolonged PT or aPTT who have a DOAC level ≥30 ng/mL; Positive likelihood ratio (LR+): Ratio of the probability of the outcome of a DOAC level <30 ng/mL over the probability of an outcome of a DOAC level ≥30 ng/mL with a normal PT or aPTT; Negative likelihood ratio (LR-): Ratio of the probability of the outcome of a DOAC level < 30 ng/mL over the probability of an outcome of a DOAC level ≥ 30 ng/mL with a prolonged PT or aPTT

**DOAC**		**Sensitivity** (95% CI)	**Specificity** (95% CI)	**PPV** (95% CI)	**NPV** (95% CI)	**LR+** (95% CI)	**LR-** (95% CI)
Overall	**PT**, s	64.3 (57.5 to 70.5)	40.0 (19.8 to 64.3)	93.7 (88.4 to 96.6)	7.5 (3.5 to 15.4)	1.1 (0.7 to 1.6)	0.9 (0.5 to 1.7)
	**aPTT**, s	78.4 (72.4 to 83.4)	46.7 (24.8 to 69.9)	95.4 (91.2 to 97.7)	13.2 (6.5 to 24.8)	1.4 (0.9 to 2.4)	0.5 (0.3 to 0.8)
Apixaban	**PT**, s	63.3 (52.3 to 73.1)	40.0 (16.8 to 68.7)	89.3 (78.5 to 95.0)	12.1 (4.8 to 27.3)	1.1 (0.6 to 1.8)	0.9 (0.4 to 2.1)
	**aPTT**, s	84.1 (74.7 to 90.5)	20.0 (5.7 to 51.0)	89.6 (80.8 to 94.6)	13.3 (3.7 to 37.9)	1.1 (0.8 to 1.5)	0.8 (0.2 to 3.0)
Dabigatran	**PT**, s	60.0 (45.5 to 73.0)	50.0 (9.5 to 90.5)	96.4 (82.8 to 99.4)	5.3 (1.0 to 24.6)	1.2 (0.3 to 4.8)	0.8 (0.2 to 3.3)
	**aPTT**, s	70.2 (56.0 to 81.3)	100.0 (34.2 to 100.0)	100.0 (89.6 to 100.0)	12.5 (3.5 to 36.0)	Inf (NA to Inf)	0.1 (0.03 to 0.4)
Rivaroxaban	**PT**, s	67.5 (56.8 to 76.6)	33.3 (6.1 to 79.2)	96.6 (88.3 to 99.0)	3.6 (0.6 to 17.7)	1.0 (0.4 to 2.3)	1.0 (0.2 to 5.0)
	**aPTT**, s	77.4 (67.4 to 85.0)	100.0 (43.9 to 100.0)	100.0 (94.4 to 100.0)	13.6 (4.7 to 33.)	Inf (NA to Inf)	0.2 (0.2 to 0.3)

DOAC levels were missing for 7 patients, aPTT for 6 patients, PT for 10 patients (due to issue with blood collection or processing); PT, prothrombin time; aPTT, activated thromboplastin time; DOAC, directed oral anticoagulants; PPV, positive predictive value; NPV, negative predictive value; LR−, negative likelihood ratio; LR+, positive likelihood ratio; 95% CI, 95% confidence interval

© 2026 Camilleri E et al. *JAMA Network Open.*

eTable 6. Sensitivity and specificity of PT and aPTT for DOAC levels <30 ng/mL, stratified by center of inclusion

Sensitivity: Proportion of patients with DOAC levels <30 ng/mL with normal PT or aPTT; Specificity: Proportion of patients with DOAC levels ≥30 ng/mL with a prolonged PT or aPTT; Positive predictive value (PPV): Proportion of patients with a normal PT or aPTT who have a DOAC level <30 ng/mL; Negative predictive value (NPV): Proportion of patients with a prolonged PT or aPTT who have a DOAC level ≥30 ng/mL; Positive likelihood ratio (LR+): Ratio of the probability of the outcome of a DOAC level <30 ng/mL over the probability of an outcome of a DOAC level ≥30 ng/mL with a normal PT or aPTT; Negative likelihood ratio (LR-): Ratio of the probability of the outcome of a DOAC level < 30 ng/mL over the probability of an outcome of a DOAC level ≥ 30 ng/mL with a prolonged PT or aPTT.

**DOAC**		**Sensitivity** (95% CI)	**Specificity** (95% CI)	**PPV** (95% CI)	**NPV** (95% CI)	**LR+** (95% CI)	**LR-** (95% CI)
**LUMC**							
Overall	**PT**, s	54.1 (46.3 to 61.7)	60.0 (31.3 to 83.2)	95.5 (89.0 to 98.2)	7.7 (3.6 to 15.8)	1.4 (0.6 to 2.9)	0.8 (0.4 to 1.3)
	**aPTT**, s	72.0 (64.5 to 78.4)	50 (23.7 to 76.3)	95.8 (90.5 to 98.3	10.2 (4.4 to 21.8)	1.4 (0.8 to 2.7)	0.6 (0.3 to 1.1)
Apixaban	**PT**, s	51.7 (39.2 to 64.1)	57.1 (25.0 to 84.2)	90.9 to (76.4 to 96.9)	12.6 (5.0 to 28.1)	1.2 (0.5 to 2.9)	0.8 (0.4 to 1.7)
	**aPTT**, s	81.0 (79.1 to 89.1)	28.6 (82.0 to 64.1)	90.4 (79.4 to 95.8)	15.4 (4.3 to 42.2)	1.1 (0.7 to 1.8)	0.7 (0.2 to 2.4)
Dabigatran	**PT**, s	50.0 (34.1 to 65.9)	50.0 (9.5 to 90.5)	94.4 (74.2 to 99.0)	5.6 (1.0 to 25.8)	1.0 (0.2 to 4.2)	1.0 (0.2 to 4.2)
	**aPTT**, s	58.8 (42.2 to 73.6)	100.0 (34.2 to 100.0)	100.0 (83.9 to 100.0)	12.5 (3.5 to 36.0)	Inf (NA to Inf)	0.4 (0.3 to 0.6)
Rivaroxaban	**PT**, s	58.5 (46.3 to 69.6)	100.0 (20.7 to 100.0)	100.0 (90.8 to 100.0)	3.6 (0.6 to 17.7)	Inf (NA to Inf)	0.4 (0.3 to 0.6)
	**aPTT**, s	70.8 (58.8 to 80.4)	100.0 (20.7 to 100.0)	100.0 (92.3 to 100.0)	5.0 (0.9 to 23.6)	Inf (NA to Inf)	0.3 (0.2 to 0.4)
**Haga**							
Overall	**PT**, s	96.4 (87.7 to 99.0)	0.0 (0.0 to 43.4)	90.9 (79.7 to 95.9)	0 (0 to 65.8)	1.0 (0.9 to 1.0)	NA (NA to Inf)
	**aPTT**, s	96.4 (87.9 to 99.0)	40.0 (11.8 to 76.9)	94.7 (85.6 to 98.2)	50 (15.0 to 85.0)	1.6 (0.8 to 3.2)	0.1 (0.02 to 0.5)
Apixaban	**PT**, s	95.2 (77.3 to 99.2)	0.0 (0.0 to 56.1)	87.0 (67.9 to 95.5)	0 (0 to 79.3)	1.0 (0.9 to 1.0)	Inf (NA to Inf)
	**aPTT**, s	91.7 (74.2 to 97.7)	0.0 (0.0 to 56.1)	88.0 (70.0 to 95.8)	0.0 (0.0 to 65.8)	0.9 (0.8 to 1.0)	Inf (NA to Inf)
Dabigatran	**PT**, s	90.9 (62.3 to 98.4)	NA (NA to NA)	100.0 (72.2 to 100.0)	NA (NA to NA)	NA (NA to NA)	NA (NA to NA)
	**aPTT**, s	100.0 (77.2 to 100.0)	NA (NA to NA)	100.0 (77.2 to 100.0)	NA (NA to NA)	NA (NA to NA)	NA (NA to NA)
Rivaroxaban	**PT**, s	100.0 (82.4 to 100.0)	0.0 (0.0 to 65.8)	90.0 (69.9 to 97.2)	NA (NA to NA)	1.0 (1.0 to 1.0)	NA (NA to NA)
	**aPTT**, s	100.0 (83.2 to 100.0)	100 (34.2 to 100.0)	100.0 (83.2 to 100.0)	100.0 (34.2 to 100.0)	Inf (NA to Inf)	0.0 (0.0 to NA)

DOAC levels were missing for 7 patients, aPTT for 6 patients, PT for 10 patients (due to issue with blood collection or processing); PT, prothrombin time; aPTT, activated thromboplastin time; DOAC, directed oral anticoagulants; PPV, positive predictive value; NPV, negative predictive value; LR−, negative likelihood ratio; LR+, positive likelihood ratio; 95% CI, 95% confidence interval, LUMC, Leiden University Medical Center; Haga, Haga Teaching Hospital

© 2026 Camilleri E et al. *JAMA Network Open.*

eTable 7. DOAC levels of patients experiencing a major bleeding during follow-up

Age	Sex	eGFR (mL/min)	Surgical specialty	Bleeding risk of surgery	DOAC type	DOAC indication	DOAC levels (ng/mL)	Type of major bleeding
79	Female	83	Gynecology	High	Apixaban	AF	7.5	Bleeding requiring reoperation
80	Male	30	Gastrointestinal surgery	High	Apixaban	AF	9.9	Fall in Hb levels ≥2.0 g/dL
64	Male	90	Gastrointestinal surgery	High	Apixaban	AF	0.7	Hemorrhagic shock due to hematemesis
69	Female	89	Gastrointestinal surgery	High	Apixaban	AF	5.2	Fall in Hb levels ≥2.0 g/dL
80	Male	64	Gastrointestinal surgery	High	Rivaroxaban	AF	0.6	Fall in Hb levels ≥2.0 g/dL
78	Female	74	Orthopedic	High	Rivaroxaban	AF	0	Fall in Hb levels ≥2.0 g/dL
70	Male	75	Urology	High	Dabigatran	AF	4.3	Fall in Hb levels ≥2.0 g/dL
71	Female	53	Gynecology	High	Dabigatran	AF	0	Fall in Hb levels ≥2.0 g/dL
65	Female	78	Gynecology	High	Dabigatran	AF	19.3	Fall in Hb levels ≥2.0 g/dL
80	Female	72	Gynecology	Moderate	Dabigatran	AF	27.4	Bleeding requiring reoperation
62	Male	83	General surgery	High	Dabigatran	VTE	0	Bleeding requiring reoperation
80	Female	62	Gastrointestinal surgery	High	Dabigatran	AF	9.0	Fall in Hb levels ≥2.0 g/dL

AF = atrial fibrillation, eGFR = estimated glomerular filtration rate, calculated with the CDK EPI formula, DOAC = direct oral anticoagulant, VTE = venous thromboembolism

© 2026 Camilleri E et al. *JAMA Network Open.*

eTable 8. Sensitivity analysis on preprocedural DOAC levels (using an alternative cut-off of 15 ng/mL) and associated surgical blood loss and postoperative complications

		All procedures		Moderate bleeding risk		High bleeding risk	
		DOAC levels		DOAC levels		DOAC levels	
		<15 ng/mL	≥15 ng/mL	<15 ng/mL	≥15 ng/mL	<15 ng/mL	≥15 ng/mL
**Surgical blood loss**	**mL, median (range)**	0 (0 to 4250)	0 (0 to 1000)	0 (0 to 300)	0 (0 to 1000)	0 (0 to 4250)	0 (0 to 720)
	**beta (95% CI)**	Ref.	-128.1 (-268.4 to 12.2)	Ref.	108.0 (-1.5 to 217.0)	Ref.	-189.2 (384.3 to 5.9)
	**beta[a] (95% CI)**	Ref.	-96.8 (-246.5 to 52.9)	-	-	-	-
	**any, n, %**	72 (36.5)	14 (26.4)	5 (22.7)	9 (39.1)	67 (38.3)	5 (16.7)
	**RR (95% CI)**	Ref.	0.7 (0.4 to 1.2)	Ref.	2.2 (0.6 to 8.6)	Ref.	0.3 (0.1 to 0.8)
	**RR[a] (95% CI)**	Ref.	0.6 (0.3 to 2.2)	-	-	-	-
**All bleedings***	**n, %**	27 (13.7)	8 (15.1)	1 (4.5)	4 (17.3)	26 (14.8)	4 (13.3)
	**RR (95% CI)**	Ref.	1.1 (0.5 to 2.3)	Ref.	3.8 (0.5 to 31.6)	Ref.	0.9 (0.3 to 2.3)
**Major bleedings***	**n, %**	10 (5.1)	2 (3.8)	0 (0)	1 (4.3)	10 (5.7)	1 (3.3)
	**RR (95% CI)**	Ref.	0.7 (0.2 to 3.3)	Ref.	NA	Ref.	0.6 (0.1 to 4.3)
**Infections***	**n, %**	26 (13.2)	4 (7.5)	1 (4.5)	1 (4.5)	25 (14.3)	3 (10.0)
	**RR (95% CI)**	Ref.	0.6 (0.2 to 1.6)	Ref.	0.9 (0.1 to 14.4)	Ref.	0.7 (0.2 to 2.2)
**Reoperation***	**n, %**	16 (8.1)	4 (7.5)	1 (4.5)	2 (8.7)	15 (8.5)	2 (6.7)
	**RR (95% CI)**	Ref.	0.9 (0.3 to 2.7)	Ref.	1.9 (0.2 to 19.6)	Ref.	0.8 (0.2 to 3.2)

*Occurring in the 30-days of follow-up. [a] From a model adjusted for the surgical bleeding risk (high or moderate). DOAC = direct oral anticoagulants, n = number, % = percentage, RR = risk ratios, CI = confidence interval, NA = not applicable

© 2026 Camilleri E et al. *JAMA Network Open.*

eTable 9. Sensitivity analysis on preprocedural DOAC levels (using an alternative cut-off of 50 ng/mL) and associated surgical blood loss and postoperative complications

		All procedures		Moderate bleeding risk		High bleeding risk	
		DOAC levels		DOAC levels		DOAC levels	
		<50 ng/mL	≥50 ng/mL	<50 ng/mL	≥50 ng/mL	<50 ng/mL	≥50 ng/mL
**Surgical blood loss**	**mL, median (range)**	0 (0 to 4250)	0 (0 to 480)	0 (0 to 1000)	0 (0 to 480)	0 (0 to 4250)	NA
	**beta (95% CI)**	Ref.	-84.9 (410.4 to 240.5)	Ref.	30.0 (-118.8 to 178.6)	Ref.	NA
	**beta[a] (95% CI)**	Ref.	29.9 (-321.9 to -381.8)	-	-	-	-
	**any, n, %**	83 (34.3)	3 (37.5)	11 (29.7)	3 (37.5)	72 (35.1)	0 (0)
	**RR (95% CI)**	Ref.	1.1 (0.4 to 2.7)	Ref.	1.4 (0.3 to 6.9)	Ref.	NA
	**RR[a] (95% CI)**	Ref.	1.4 (0.3 to 2.8)	-	-	-	-
**All bleedings***	**n, %**	34 (14.0)	1 (13.0)	4 (10.8)	1 (12.5)	30 (14.6)	0 (0)
	**RR (95% CI)**	Ref.	0.9 (0.1 to 5.7)	Ref.	1.2 (0.1 to 9.0)	Ref.	NA
**Major bleedings***	**n, %**	12 (5.0)	0 (0)	1 (2.7)	0 (0)	11 (5.4)	0 (0)
	**RR (95% CI)**	Ref.	NA	Ref.	NA	Ref.	NA
**Infections***	**n, %**	30 (12.4)	0 (0)	2 (5.4)	0 (0)	28 (13.7)	0 (0)
	**RR (95% CI)**	Ref.	NA	Ref.	NA	Ref.	NA
**Reoperation***	**n, %**	20 (8.3)	0 (0)	3 (8.1)	0 (0)	17 (8.2)	0 (0)
	**RR (95% CI)**	Ref.	NA	Ref.	NA	Ref.	NA

*Occurring in the 30-days of follow-up. [a] From a model adjusted for the surgical bleeding risk (high or moderate). DOAC = direct oral anticoagulants, n = number, % = percentage, RR = risk ratios, CI = confidence interval, NA = not applicable

© 2026 Camilleri E et al. *JAMA Network Open.*

eFigure 1. Sensitivity analysis on the proportion of preoperative DOAC levels ≥15 ng/mL overall (A) and by specific DOAC (B), shown for all procedures and stratified by bleeding risk of the procedures

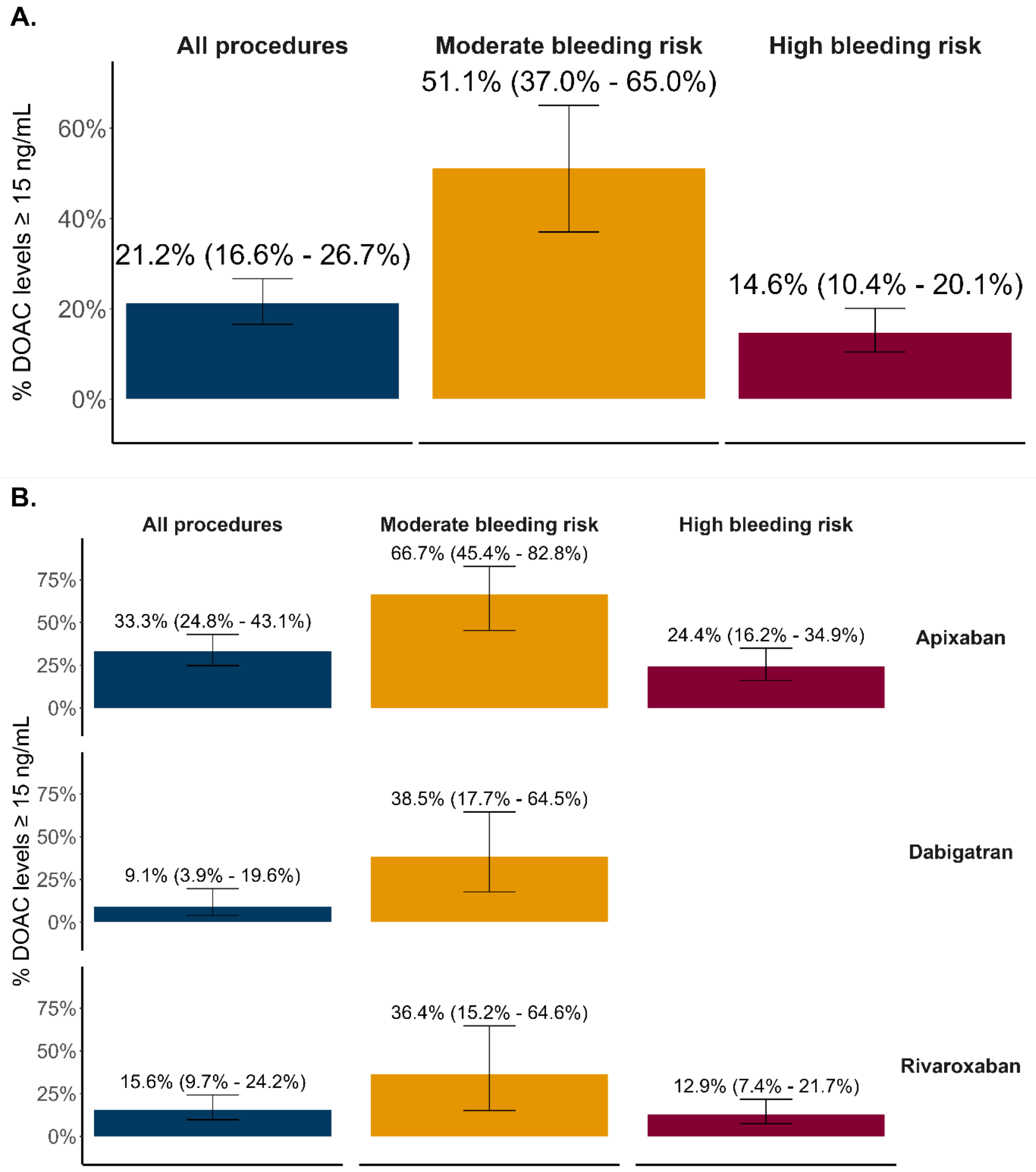

© 2026 Camilleri E et al. *JAMA Network Open.*

eFigure 2. Sensitivity analysis on the proportion of preoperative DOAC levels ≥50 ng/mL overall (A) and by specific DOAC (B), shown for all procedures and stratified by bleeding risk of the procedures

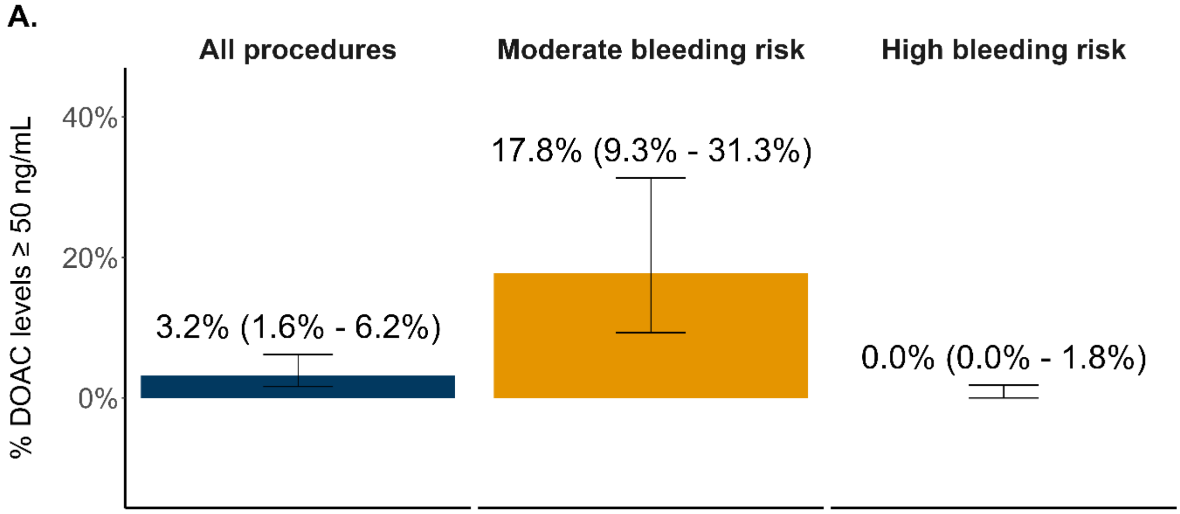

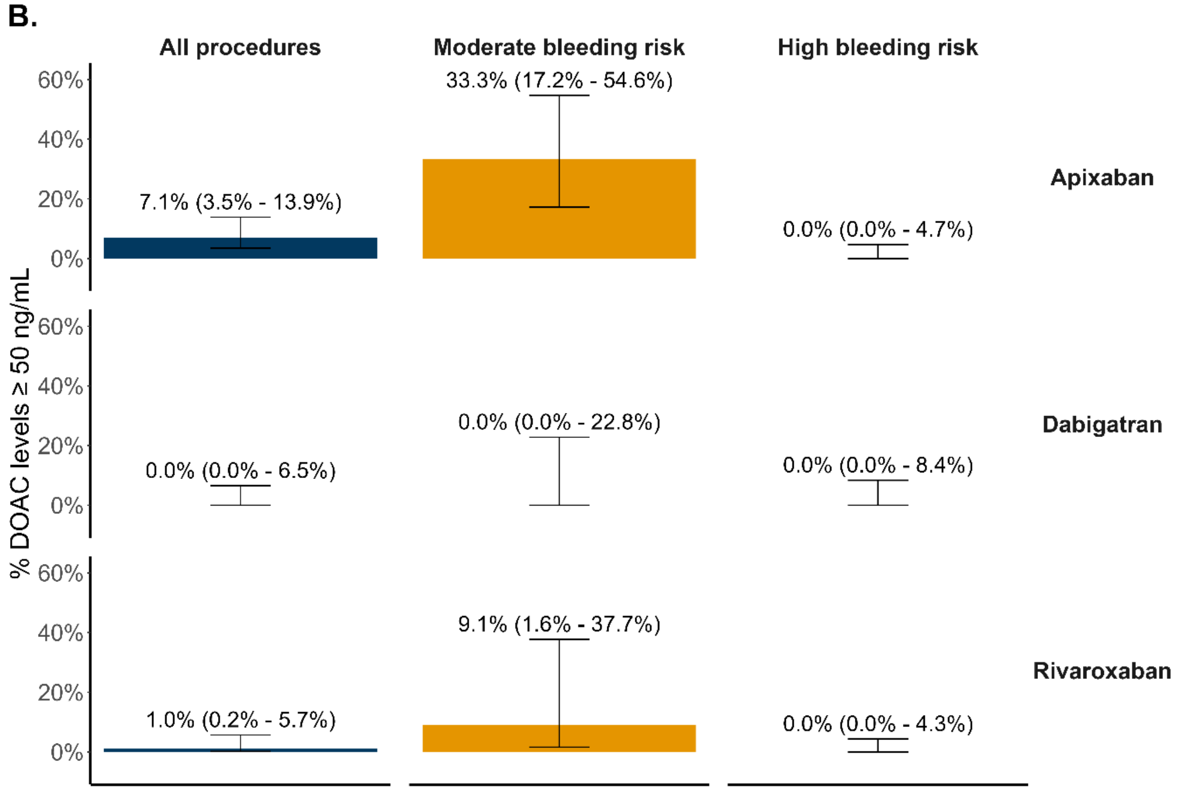

© 2026 Camilleri E et al. *JAMA Network Open.*

eFigure 3. Proportion of preoperative prolonged PT and aPTT levels for all DOACs (A) and stratified by DOAC type (A)

PT is depicted in blue, aPTT in red. PT was considered prolonged when ≥14.9s for patients included at the LUMC and if ≥12.0 s for patients included at the Haga Teaching Hospital, aPTT when ≥31.7 s. PT and aPTT were missing due to issues due to technical issues during blood collection or processing in 10 and 6 patients respectively. %, percentage, PT, prothrombin time; aPTT, activated thromboplastin time; DOAC, directed oral anticoagulants, LUMC Leiden University Medical Center.

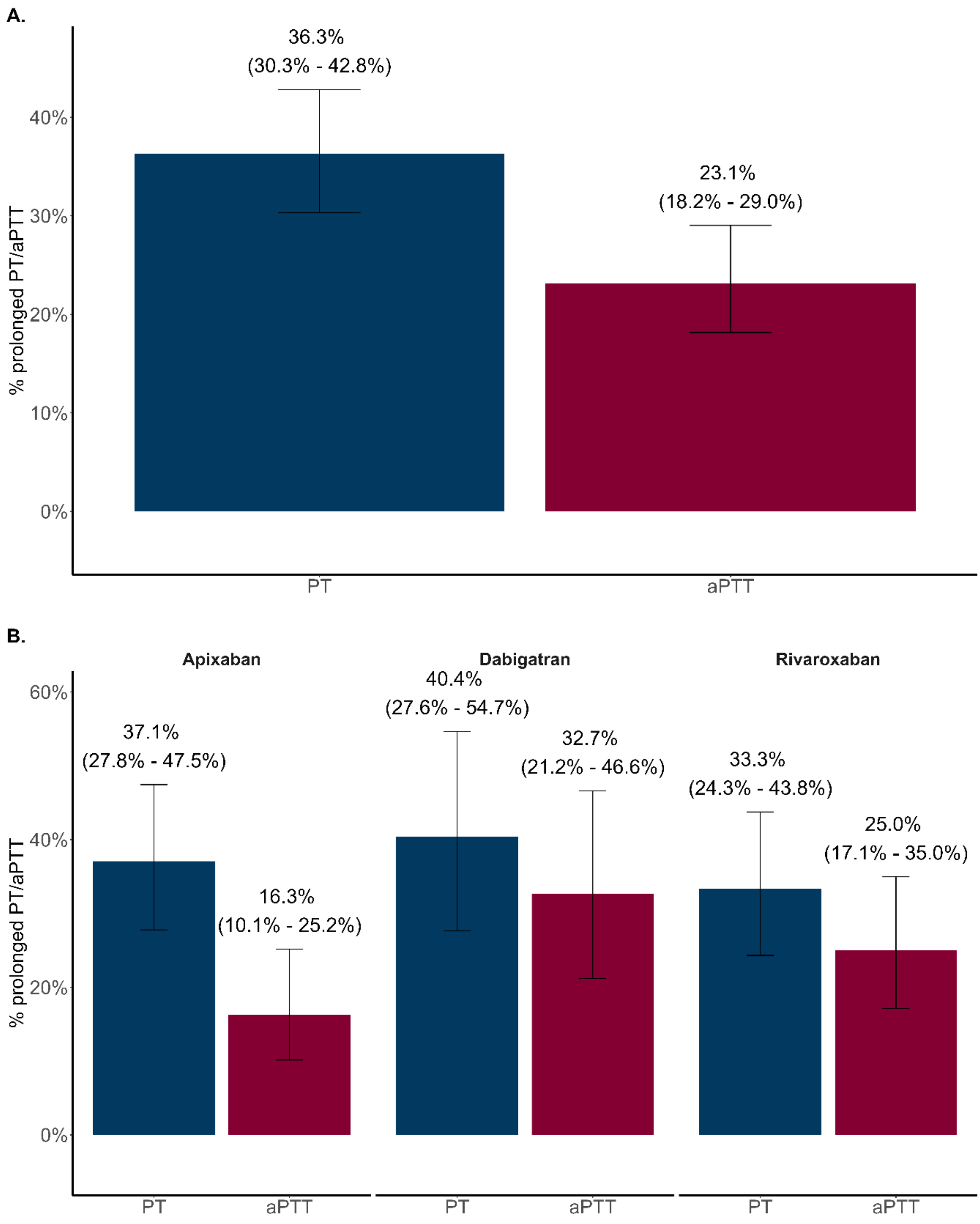

© 2026 Camilleri E et al. *JAMA Network Open.*

eFigure 4. Correlation between preoperative PT and DOAC levels (A. all DOACs, B. stratified by DOAC type)

The vertical red dotted line represents the cut-off for elevated PT (≥14.9 s for patients included at the LUMC and if ≥12.0 s for patients included at the Haga Teaching Hospital) and the horizontal dotted line for elevated DOAC levels (≥30 ng/mL). PT, prothrombin time; DOAC, directed oral anticoagulants ρ, Spearman rank correlation coefficient.

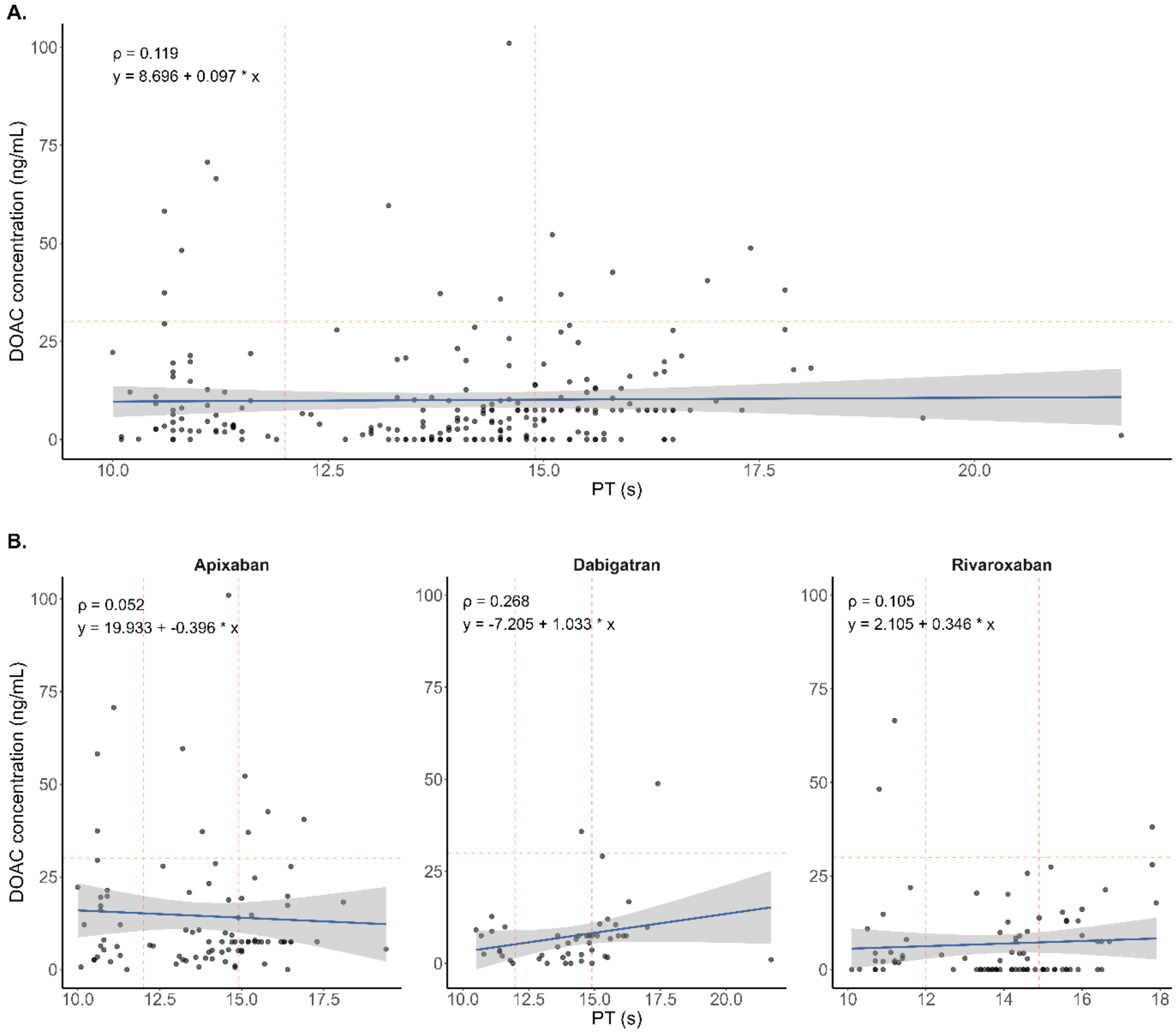

© 2026 Camilleri E et al. *JAMA Network Open.*

eFigure 5. Correlation between preoperative aPTT and DOAC levels (A. all DOACs, B. stratified by DOAC type)

The vertical red dotted line represents the cut-off for elevated aPTT (≥31.7 s for both centers) and the horizontal dotted line for elevated DOAC levels (≥30 ng/mL). aPTT, activated thromboplastin time; DOAC, directed oral anticoagulants ρ, Spearman rank correlation coefficient.

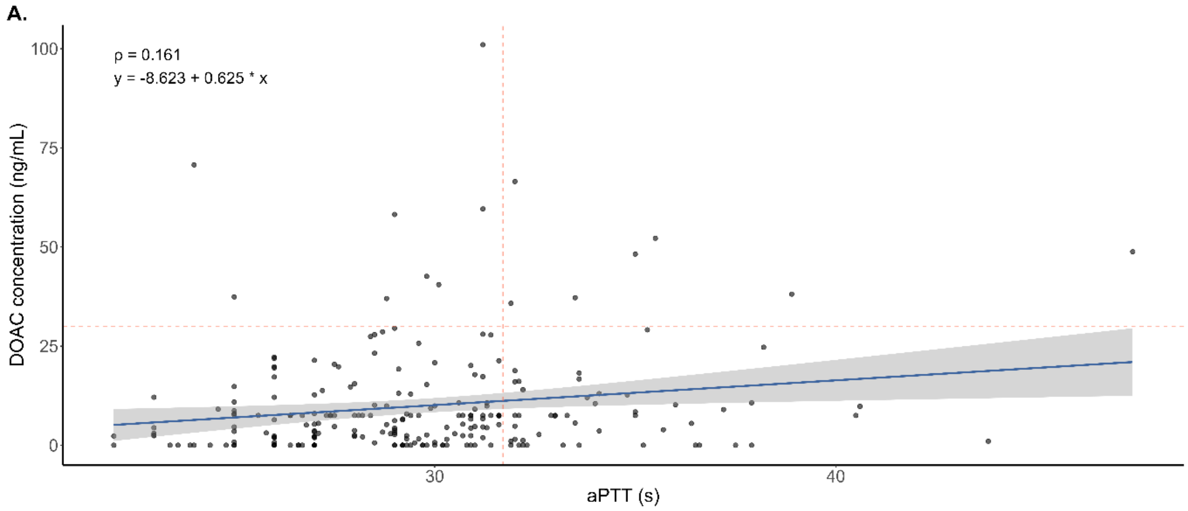

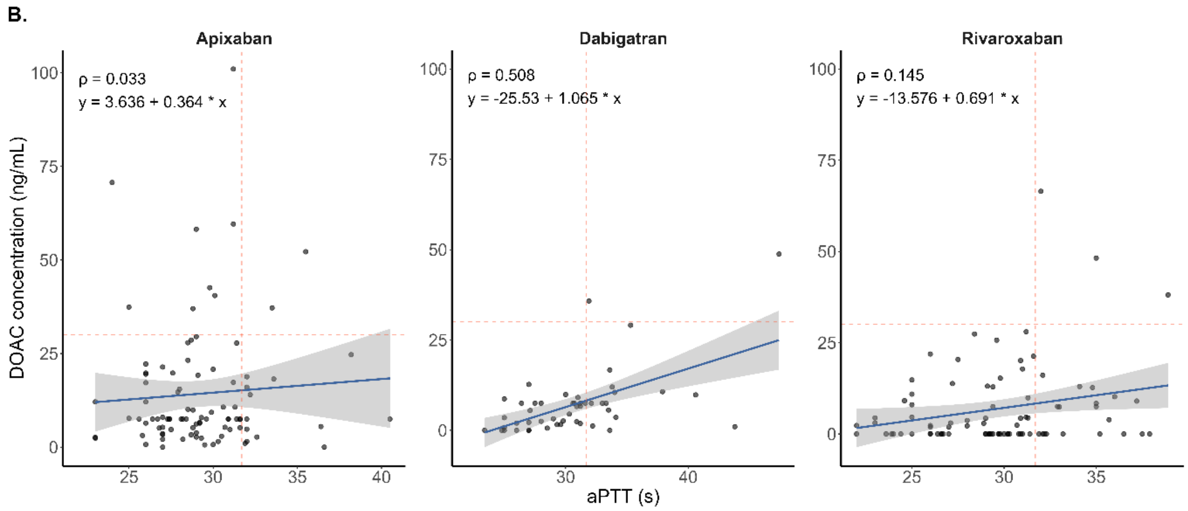

© 2026 Camilleri E et al. *JAMA Network Open.*

eFigure 6. Correlation between preoperative PT and DOAC levels, stratified by center (A. all DOACs, B. stratified by DOAC type)

The vertical red dotted line represents the cut-off for elevated PT (≥14.9 s for patients included at the LUMC and if ≥12.0 s for patients included at the Haga Teaching Hospital) and the horizontal dotted line for elevated DOAC levels (≥30 ng/mL). PT, prothrombin time; DOAC, directed oral anticoagulants ρ, Spearman rank correlation coefficient; LUMC, Leiden University Medical Center; Haga, Haga Teaching Hospital.

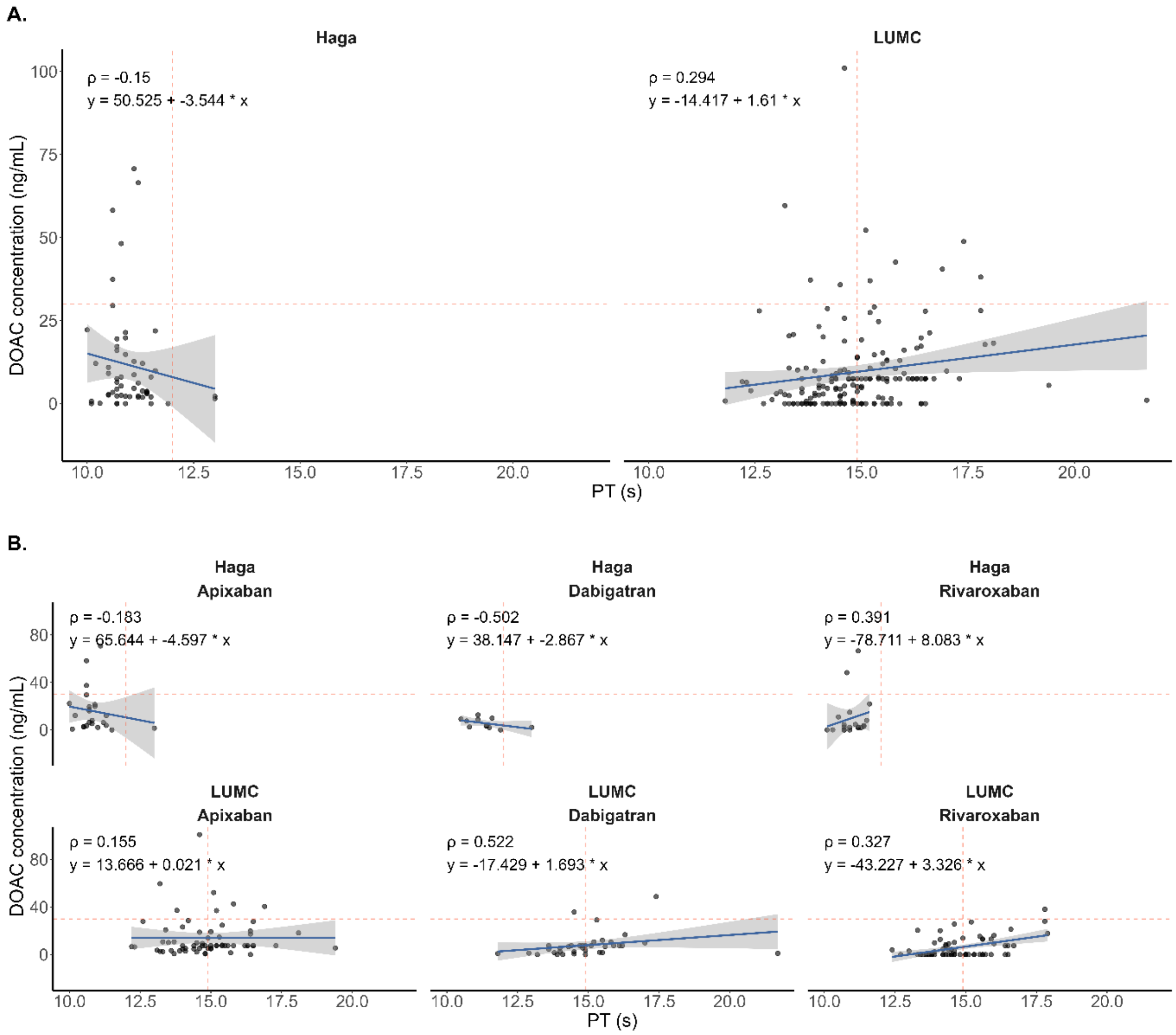

© 2026 Camilleri E et al. *JAMA Network Open.*

eFigure 7. Correlation between preoperative aPTT and DOAC levels, stratified by center (A. all DOACs, B. stratified by DOAC type)

The vertical red dotted line represents the cut-off for elevated aPTT (≥31.7 for both centers) and the horizontal dotted line for elevated DOAC levels (≥30 ng/mL). aPTT, activated thromboplastin time; DOAC, directed oral anticoagulants ρ, Spearman rank correlation coefficient; LUMC, Leiden University Medical Center; Haga, Haga Teaching Hospital.

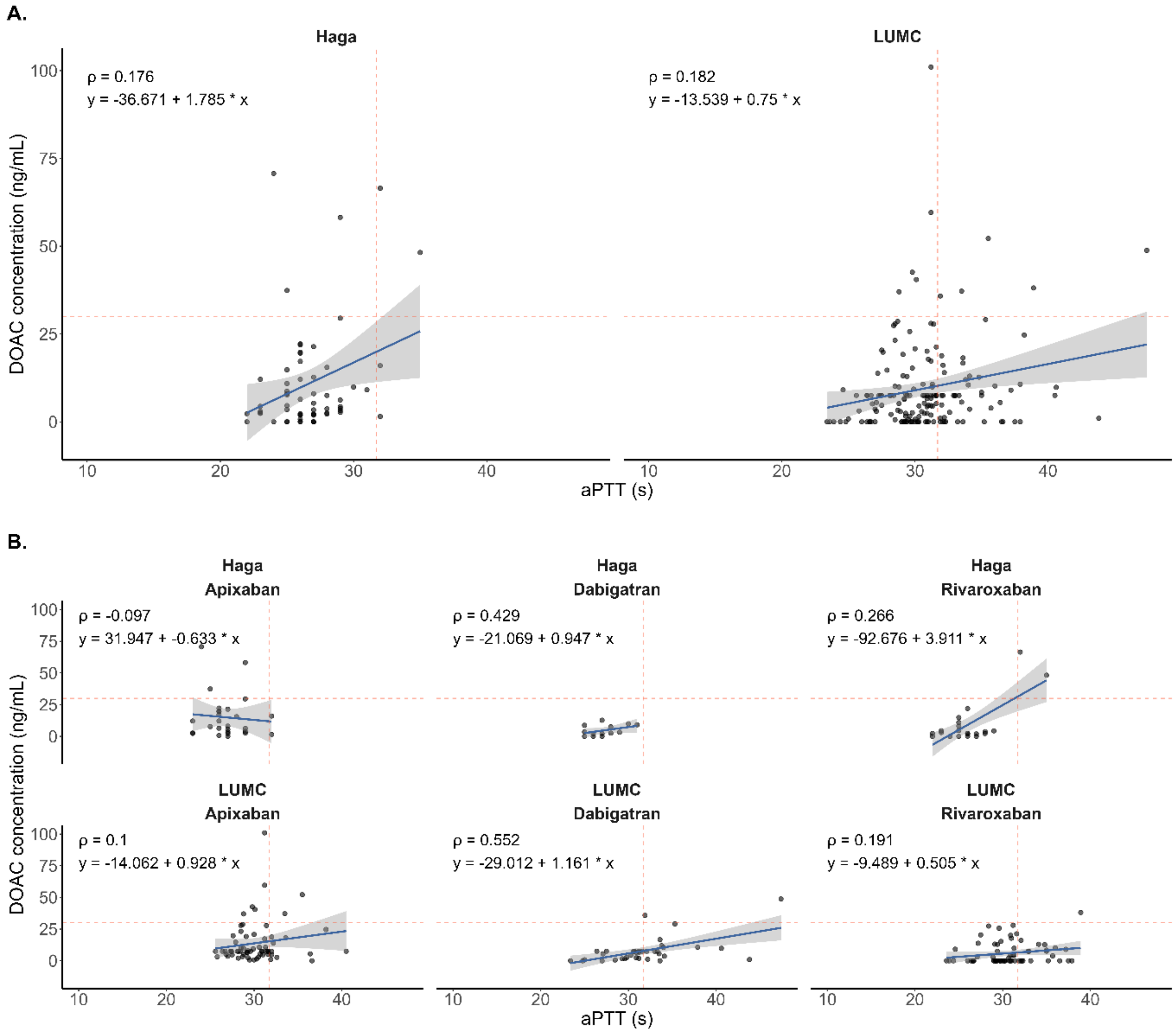

© 2026 Camilleri E et al. *JAMA Network Open.*
