# Supplementary material for: Direct Oral Anticoagulant Levels at Time of Elective Surgery
Source: JAMA Netw Open. 2026 Feb 4;9(2):e2555875. doi: 10.1001/jamanetworkopen.2025.55875 (PMC12873767; doi:10.1001/jamanetworkopen.2025.55875)
Supplement: Supplement 2. — Data Sharing Statement [file jamanetwopen-e2555875-s002.pdf]

Data Sharing Statement

Camilleri. Direct Oral Anticoagulant Levels at Time of Elective Surgery. *JAMA Netw Open*.
Published February 04, 2026. doi:10.1001/jamanetworkopen.2025.55875

Data
Data available: No

Additional Information
Explanation for why data not available: The data that support the findings of this study are available from the corresponding author upon reasonable request.
